# Novel sarbecovirus bispecific neutralizing antibodies with exceptional breadth and potency against currently circulating SARS-CoV-2 variants and sarbecoviruses

**DOI:** 10.1038/s41421-022-00401-6

**Published:** 2022-04-21

**Authors:** Yingdan Wang, Meiqin Liu, Yaping Shen, Yunping Ma, Xiang Li, Yuanyuan Zhang, Mei Liu, Xing-Lou Yang, Jun Chen, Renhong Yan, Die Luan, Yanqun Wang, Ying Chen, Qimin Wang, Haofeng Lin, Yaning Li, Kaiyue Wu, Tongyu Zhu, Jincun Zhao, Hongzhou Lu, Yumei Wen, Shibo Jiang, Fan Wu, Qiang Zhou, Zheng-Li Shi, Jinghe Huang

**Affiliations:** 1 Key Laboratory of Medical Molecular Virology (MOE/NHC/CAMS) and Shanghai Institute of Infectious Disease and Biosecurity, Shanghai Public Health Clinical Center, School of Basic Medical Sciences, Fudan University, Shanghai, China; 2 CAS Key Laboratory of Special Pathogens, Wuhan Institute of Virology, Center for Biosafety Mega-Science, Chinese Academy of Sciences, Wuhan, Hubei China; 3 University of Chinese Academy of Sciences, Beijing, China; 4 Center for Infectious Disease Research, Westlake Laboratory of Life Sciences and Biomedicine, Key Laboratory of Structural Biology of Zhejiang Province, School of Life Sciences, Westlake University, Hangzhou, Zhejiang China; 5 Institute of Biology, Westlake Institute for Advanced Study, Hangzhou, Zhejiang China; 6 Shanghai Immune Therapy Institute, Shanghai Jiao Tong University School of Medicine Affiliated Renji Hospital, Shanghai, China; 7 State Key Laboratory of Respiratory Disease, National Clinical Research Center for Respiratory Disease, Guangzhou Institute of Respiratory Health, the First Affiliated Hospital of Guangzhou Medical University, Guangzhou, Guangdong China; 8 Institute of Infectious Disease, Guangzhou Eighth People’s Hospital of Guangzhou Medical University, Guangzhou, Guangdong China; 9 Beijing Advanced Innovation Center for Structural Biology, Tsinghua-Peking Joint Center for Life Sciences, School of Life Sciences, Tsinghua University, Beijing, China

**Keywords:** Mechanisms of disease, Cryoelectron microscopy, Autoimmunity

## Abstract

The emergence of severe acute respiratory syndrome coronavirus-2 (SARS-CoV-2) variants of concern, including Alpha (B.1.1.7), Beta (B.1.351), Gamma (P.1), Delta (B.1.617.2), and Omicron (B.1.1.529) has aroused concerns over their increased infectivity and transmissibility, as well as decreased sensitivity to SARS-CoV-2-neutralizing antibodies (NAbs) and the current coronavirus disease 2019 (COVID-19) vaccines. Such exigencies call for the development of pan-sarbecovirus vaccines or inhibitors to combat the circulating SARS-CoV-2 NAb-escape variants and other sarbecoviruses. In this study, we isolated a broadly NAb against sarbecoviruses named GW01 from a donor who recovered from COVID-19. Cryo-EM structure and competition assay revealed that GW01 targets a highly conserved epitope in a wide spectrum of different sarbecoviruses. However, we found that GW01, the well-known sarbecovirus NAb S309, and the potent SARS-CoV-2 NAbs CC12.1 and REGN10989 only neutralize about 90% of the 56 tested currently circulating variants of SARS-CoV-2 including Omicron. Therefore, to improve efficacy, we engineered an IgG-like bispecific antibody GW01-REGN10989 (G9) consisting of single-chain antibody fragments (scFv) of GW01 and REGN10989. We found that G9 could neutralize 100% of NAb-escape mutants (23 out of 23), including Omicron variant, with a geometric mean (GM) 50% inhibitory concentration of 8.8 ng/mL. G9 showed prophylactic and therapeutic effects against SARS-CoV-2 infection of both the lung and brain in hACE2-transgenic mice. Site-directed mutagenesis analyses revealed that GW01 and REGN10989 bind to the receptor-binding domain in different epitopes and from different directions. Since G9 targets the epitopes for both GW01 and REGN10989, it was effective against variants with resistance to GW01 or REGN10989 alone and other NAb-escape variants. Therefore, this novel bispecific antibody, G9, is a strong candidate for the treatment and prevention of infection by SARS-CoV-2, NAb-escape variants, and other sarbecoviruses that may cause future emerging or re-emerging coronavirus diseases.

## Introduction

Coronaviruses are a group of diverse RNA viruses that infect a wide range of animals from bats, rodents, and birds to several domestic animals. The zoonotic spillover of coronavirus into the human population has caused three major pandemic threats to public health in the last two decades, including severe acute respiratory syndrome (SARS)^1^, Middle East respiratory syndrome (MERS)^2^ and COVID-19^3,4^. The ongoing pandemic of COVID-19, caused by SARS-CoV-2, has resulted in more than 415 million cases of infection and 5.8 million deaths as of 17 February 2022 (WHO COVID-19 DASHBOARD). No effective therapeutic drug against SARS-CoV-2 is currently available, and vaccines are considered critical to ending the pandemic. However, the emergence of SARS-CoV-2 variants of concern (VOCs), such as Alpha (B.1.1.7), Beta (B.1.351), Gamma (P.1), Delta (B.1.617.2), and Omicron (B.1.1.529)^5,6^ as well as variants of interest (VOIs), including Eta (B.1.525), Iota (B.1.526), Kappa (B.1.617.1), and Lambda (C.37), has aroused the concerns that they may escape the purported efficacy of neutralizing antibodies (NAbs), rendering the current vaccines ineffective^7^. This calls for the development of prophylactics, therapeutics, and vaccines to combat a broad-spectrum of sarbecoviruses, including SARS-CoV-2 and its variants, SARS-CoV, and SARS-related coronaviruses (SARSr-CoVs), that may cause future outbreaks of emerging or re-emerging coronavirus diseases^8^.

A rationally designed pan-sarbecovirus vaccine is expected to induce NAbs broadly against the conserved epitopes in spike (S) proteins among different sarbecoviruses^8^. SARS-CoV-2 shares 77.2% amino-acid identity in its S proteins with SARS-CoV^4^. Several NAbs isolated from SARS-CoV-infected patients, including CR3022^9^, S309^10^, CC6.33^11^, H014^12^, COVA1-16^13^, CV38-142^14^, ADG-2^15^, and S2H97^16^, showed cross-neutralization against SARS-CoV-2, suggesting the existence of conserved neutralizing epitopes in S proteins of sarbecoviruses, which could serve as a basis for the design of pan-sarbecovirus vaccines.

Many potent SARS-CoV-2-specific NAbs have already been discovered (review in ref. ^17^). Moreover, combining two NAbs^18^ or developing a bispecific NAb on the basis of two NAbs that target different neutralizing epitopes in the SARS-CoV-2 S protein^19^ showed increased therapeutic and prophylactic efficacy. However, the construction of a bispecific NAb using two highly potent NAbs targeting different neutralizing epitopes in the receptor-binding domain (RBD) with broad neutralizing activities against sarbecoviruses has not been reported thus far. Here, we used a broad sarbecovirus NAb designated GW01, which was isolated from a patient who recovered from COVID-19, and another NAb, REGN10989^18^, which targets a different neutralizing epitope from GW01, to construct a bispecific antibody, termed GW01-REGN10989 (G9). We found that G9 potently neutralized SARS-CoV-2 and its VOCs, including the Omicron variant, as well as other sarbecoviruses, such as SARS-CoV and SARSr-CoVs from bats and pangolins. The results from competition assays, cryo-EM structure analysis, and site-directed mutagenesis all revealed that GW01 binds to a conserved epitope in the RBD in the S proteins of many sarbecoviruses. G9 targets the epitopes for both GW01 and REGN10989, thus exhibiting efficacy against divergent sarbecoviruses, including variants resistant to GW01 or REGN10989 alone. Therefore, this bispecific NAb can be developed for the treatment and prevention of infection by SARS-CoV-2 and its VOCs, as well as other sarbecoviruses.

## Results

### Identification of a donor who recovered from COVID-19 with potent cross-neutralizing serum

We first screened the plasma of 245 donors who had recovered from COVID-19 for neutralizing antibodies^20^ and identified two donors whose plasma showed high titers of SARS-CoV-2 NAb. In particular, the plasma from Donor 1, a 60-year-old male who recovered from mild COVID-19, showed extremely high titers of SARS-CoV-2 NAb with a 50% neutralizing titer (NT_50_) of 52,640 and exhibited cross-neutralization against pseudotyped SARS-CoV and bat SARSr-CoV WIV1 and Rs3367^21^ (NT_50_: 2881, 1472, and 5083, respectively, Supplementary Fig. S1a). The increase in SARS-CoV-2 NAb titre (Supplementary Fig. S1b) was largely associated with the increase in RBD-binding antibodies (Supplementary Fig. S1c) in the sequentially collected plasma samples (Day 4–36). Binding antibodies that were cross-reactive with SARS-CoV RBD and S1 proteins were also detected (Supplementary Fig. S1d).

The pipeline to isolate NAbs from patients who recovered from COVID-19 is shown in Supplementary Fig. S1e. In brief, 85,000 peripheral memory B cells were sorted and cultured in a 384-well plate for two weeks as previously described^22^. Supernatants of B-cell microcultures were screened for the inhibition of pseudotyped SARS-CoV-2 and SARS-CoV infection by using a high-throughput 384-well-based microneutralization assay. Variable regions of IgG genes in positive wells were cloned and re-expressed in 293 T cells for further characterization.

### Isolation of a potent and broad sarbecovirus-neutralizing mAb GW01

The supernatants from five wells exhibited potent neutralizing activity (> 90% inhibition) against SARS-CoV-2, and two wells from Donor 1 showed cross-neutralization (> 90% inhibition) against SARS-CoV. Cloning and cotransfection of the heavy and light chain plasmids into 293 T cells resulted in the expression of IgG, termed GW01, 6I18, 3D13, 10C2, and 22H22. The mAbs GW01 and 6I18 were isolated from the two wells showing cross-neutralization activity. The germline and complementary determining region 3 (CDR3) sequences of the five mAbs are listed in Supplementary Table S1. The heavy chain of antibody GW01 was derived from IGHV3–43, and it has the longest CDR3 region of 20 amino acids. The heavy chain of antibody 6I18 was derived from IGHV4–59 with a CDR3 region of 16 amino acids. The heavy chains of the other three antibodies were derived from IGHV3–53 with CDR3 regions of 11–12 amino acids. Consistent with the previously described SARS-CoV-2 antibodies^23^, all five antibodies showed only minimal somatic mutations with mutation rates less than 5% compared with germline nucleotide sequences.

### Binding specificity and neutralization activity of GW01

**Fig. 1:**
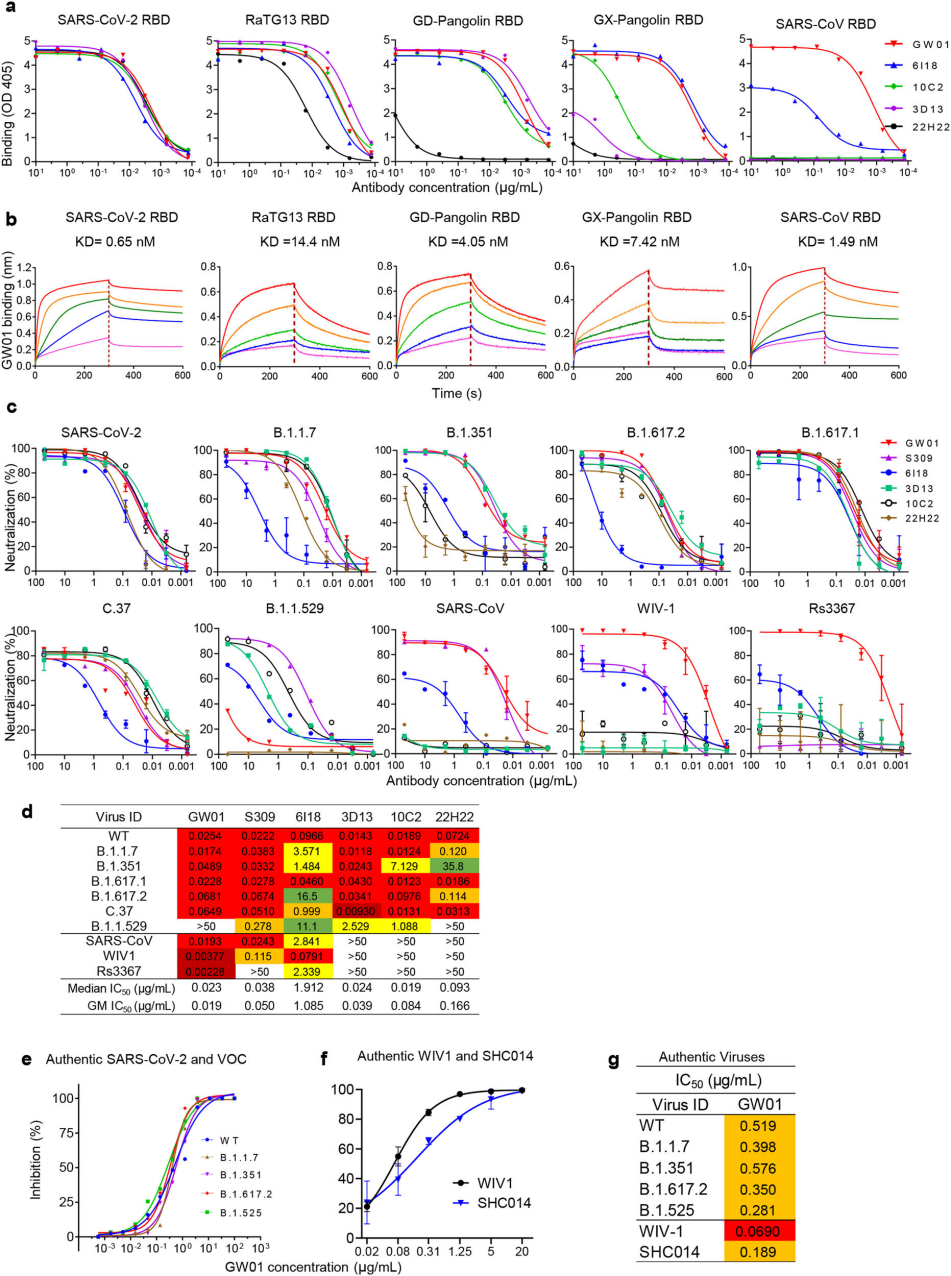
Isolation of sarbecovirus-neutralizing antibody GW01 from COVID-19 patients. **a** Binding of five mAbs isolated from COVID-19 patients to the RBD proteins of SARS-CoV-2, RaTG13, GD-Pangolin, GX-Pangolin, and SARS-CoV in ELISA. **b** Binding affinity of GW01 to RBD proteins of SARS-CoV-2, RaTG13, GD-Pangolin, GX-Pangolin, or SARS-CoV was measured by bilayer interferometry experiments. **c** Neutralization of mAbs against pseudovirus of SARS-CoV-2 and its variants B.1.1.7, B.1.351, B.1.617.1, B.1.617.2, B.1.1.529, and C.37, SARS-CoV, and bat SARSr-CoVs (WIV1 and Rs3367). **d** Neutralization summary of GW01, S309, and other NAbs against pseudoviruses. **e** Neutralization of GW01 against authentic SARS-CoV-2 and its variants B.1.1.7, B.1.351, B.1.617.2, and B.1.525. **f** Neutralization of GW01 against authentic bat SARSr-CoVs (WIV1 and SHC014). **g** IC_50_s of GW01 against seven authentic viruses.

The binding specificities of GW01, 6I18, 3D13, 10C2, and 22H22 were evaluated by enzyme-linked immunosorbent assay (ELISA) with purified SARS-CoV-2 S proteins. GW01 showed strong binding to the RBD proteins of SARS-CoV-2, RaTG13, GD-pangolin, GX-pangolin, and SARS-CoV (Fig. 1a), indicating that GW01 is a cross-binding antibody against divergent sarbecoviruses. The binding affinities of GW01 to RBD proteins of SARS-CoV-2, RaTG13, GD-pangolin, GX-pangolin, and SARS-CoV ranged from 0.65 to 14.4 nM (Fig. 1b). GW01 had a stronger binding affinity for the SARS-CoV-2 RBD than for 3D13, 10C2, or 22H22 (Supplementary Fig. S2).

The neutralization potency of these five antibodies was evaluated by using pseudotyped SARS-CoV-2 and the variants Alpha (B.1.1.7), Beta (B.1.351), Kappa (B.1.617.1), Delta (B.1.617.2), Lambda (C.37), and Omicron (B.1.1.529), as well as SARS-CoV and bat SARSr-CoV WIV1 and RS3367, on Huh-7 cells. GW01 showed neutralization potency similar to that of the previously described S309^10^ against SARS-CoV-2 and its VOCs, B.1.1.7, B.1.351, B.1.617.1, B.1.617.2, and C.37. However, GW01 did not neutralize B.1.1.529 (Fig. 1c, d). 6I18, 3D13, and 10C2 neutralized all the tested VOCs and VOIs, including B.1.1.529 (Fig. 1c, d). GW01 displayed highly potent neutralizing activity against SARS-CoV and Bat SARSr-CoV WIV1 and Rs3367 with 50% inhibitory concentration (IC_50_) values of 19.31, 3.77, and 2.28 ng/mL, respectively (Fig. 1c, d), whereas S309 potently neutralized all VOCs and SARS-CoV, as previously described, but was less potent against WIV1 and did not neutralize Rs3367.

The neutralization potency of antibody GW01 was further evaluated using a plaque reduction neutralization test in vitro on Vero-E6 cells against authentic coronaviruses. GW01 neutralized authentic SARS-CoV-2, B.1.1.7, B.1.351, B.1.617.2, and B.1.525 with IC_50_ values in the range of 0.28 to 0.57 μg/mL, indicating that GW01 is equally effective against SARS-CoV-2 WT and VOCs (Fig. 1e, g). GW01 could also potently neutralize authentic bat SARSr-CoV WIV1 and SHC014^21^ with IC_50_ values of 0.069 and 0.189 μg/mL, respectively (Fig. 1f, g). Taken together, these data indicate that GW01 is a potent mAb that neutralizes not only SARS-CoV-2 and its VOCs but also other sarbecoviruses, such as SARS-CoV and SARSr-CoVs (WIV1 and SHC014).

### GW01 binds to an RBD epitope distinct from that of REGN10989 and overlaps with the ACE2-binding site in RBD

**Fig. 2:**
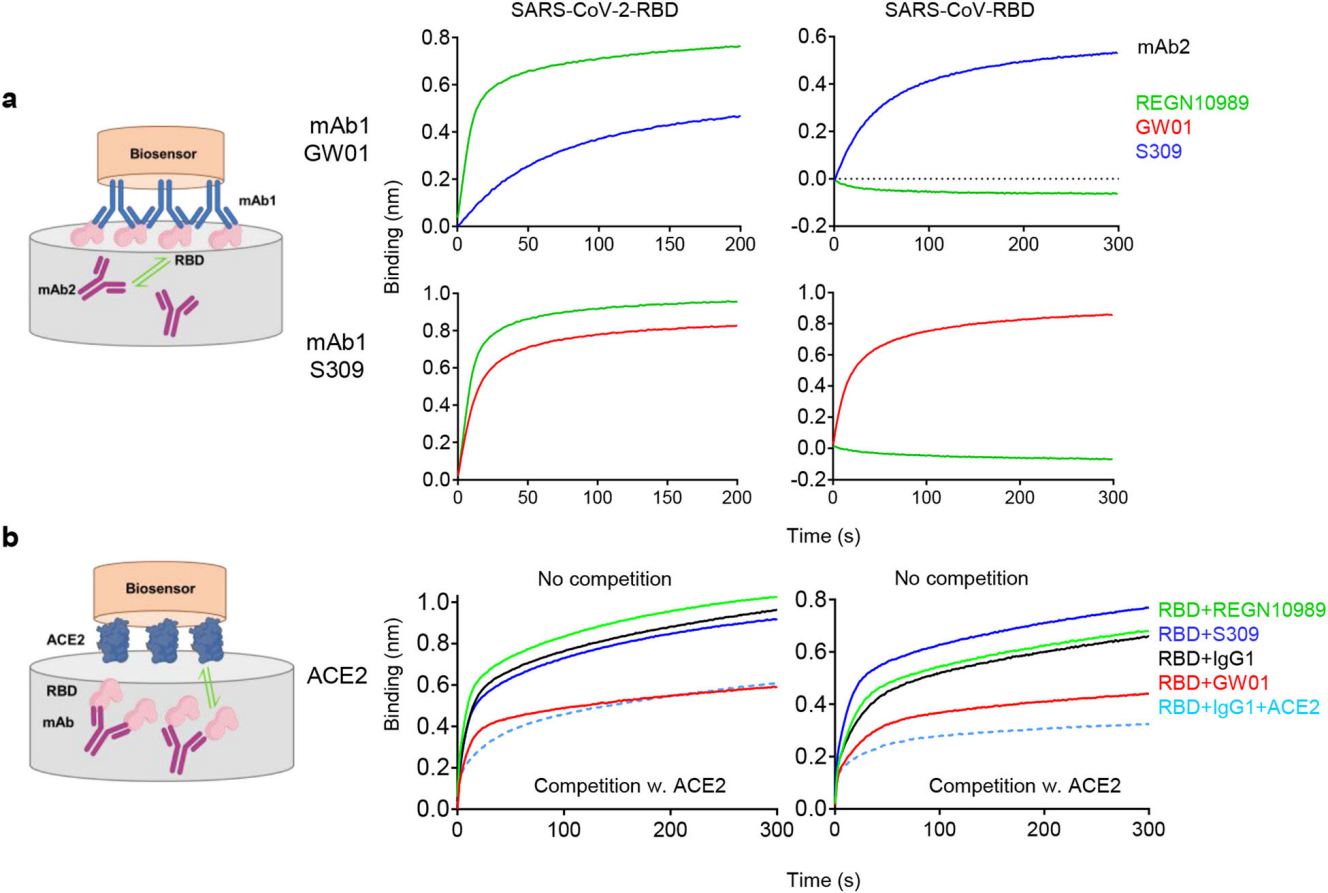
GW01 binds to an RBD epitope distinct from that of REGN10989 and overlaps with the ACE2-binding site. **a** Binding of GW01 to SARS-CoV-2 RBD (left) and SARS-CoV RBD (right) in competition with S309 and REGN10989 as measured by bilayer interferometry experiments. S309 was used as a control. **b** Binding of ACE2 to SARS-CoV-2 RBD (left) and SARS-CoV RBD (right) in competition with GW01 (red), S309 (blue), REGN10989 (green), control IgG1, and ACE2.

To determine whether GW01 binds to a domain on RBD distinct from that of REGN10989, S309, 6I18, 3D13, 10C2, or 22H22, we used bilayer interferometry experiments to test the binding of GW01 to both SARS-CoV-2 RBD and SARS-CoV RBD in competition with other antibodies. GW01 showed no competition with REGN10989, S309 (Fig. 2a), 6I18, 3D13, 10C2, or 22H22 (Supplementary Fig. S3) for RBD binding. However, GW01 competed with ACE2 for binding to SARS-CoV-2 RBD and SARS-CoV RBD, while REGN10989 and S309 did not (Fig. 2b). These results indicated that the binding epitope of GW01 is different from that of REGN10989 or S309 and that GW01 binds to an epitope that overlaps with the ACE2-binding site.

### GW01 targets a conserved RBD epitope shared by many different sarbecoviruses

**Fig. 3:**
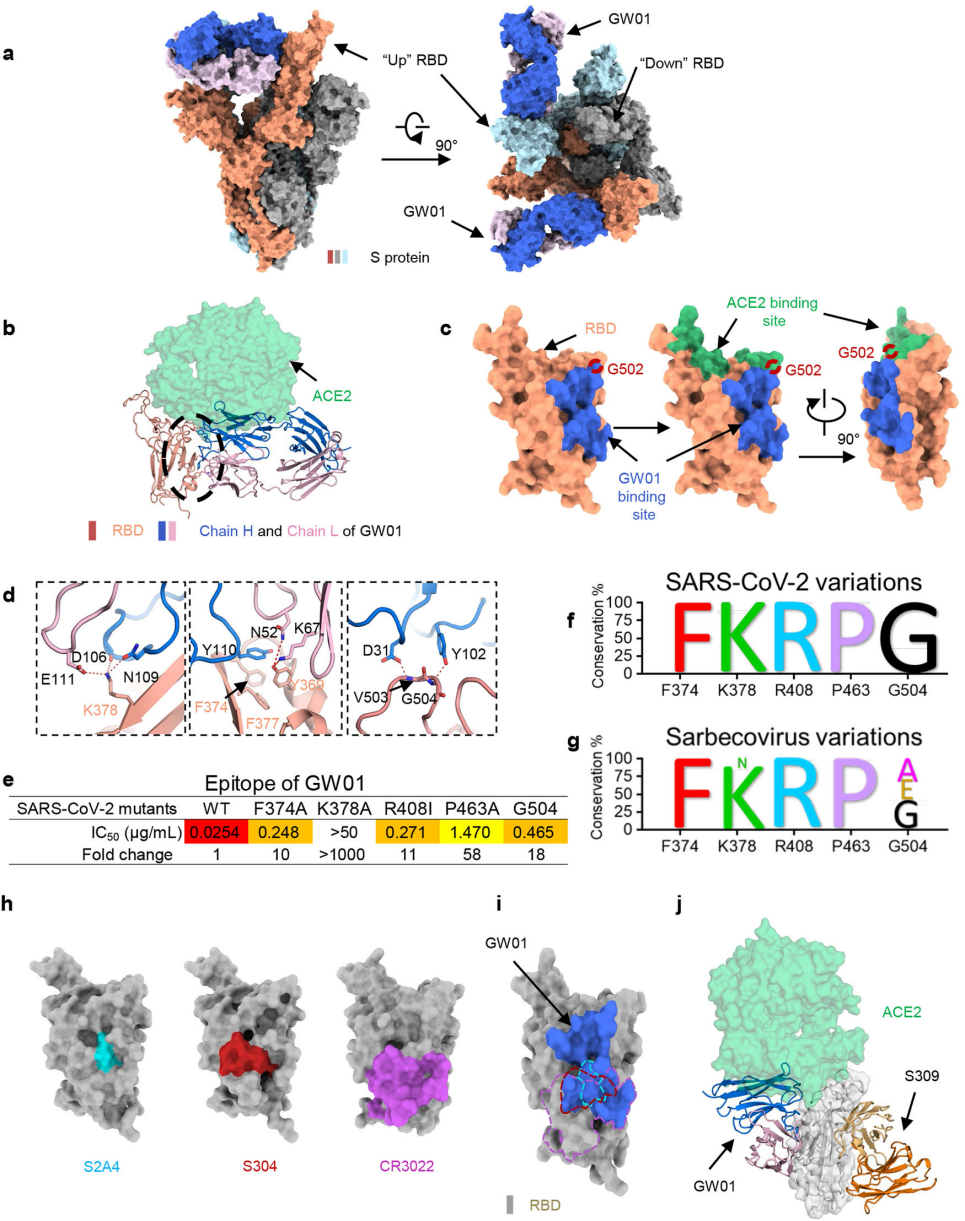
GW01 targets a conserved RBD epitope shared by the spike proteins of different sarbecoviruses. **a** The domain-colored cryo-EM structure of GW01 bound with S-ECD is shown in two perpendicular views. The heavy (blue) and light (pink) chains of GW01 bind to two “up” RBDs. Two up RBD monomers are colored orange and light blue, while the down RBD is colored gray. **b** Structural comparison of epitopes between ACE2 (green) and GW01 bound to RBD. The black circle includes extensive hydrophilic interactions on the interface between GW01 and RBD. **c** The binding epitope of GW01 bound to the RBD of S-ECD is colored blue, shown on the left, and is observed to overlap with the green-colored epitope of ACE2 bound to RBD on G502 (red ring). **d** Detailed analysis of the interface between GW01 and RBD. Polar interactions are indicated by red dashed lines. **e** Epitope of GW01. Residues that decreased neutralization sensitivities (fold change >10) of GW01. Conservation of residues F374, K378, R408, P463, and G504 in SARS-CoV-2 variant sequences (**f**) and different sarbecoviruses (**g**). **h** The RBD epitopes of S2A4 (PDB ID: 7JVC), S304 (PDB ID: 7JW0), and CR3022 (PDB ID: 6W41), which are colored cyan, red, and violet, respectively. **i** Comparison of RBD epitopes between GW01 and S2A4, S304, and CR3022. The RBD epitopes of S2A4, S304, and CR3022 are shown as meshes and are colored cyan, red, and violet, respectively. **j** Structural classification of GW01 and S309 (PDB ID: 6WPT) with no overlaps between them. In GW01, the heavy chain and light chain are shown in blue and pink, respectively, and in S309, they are shown in orange and wheat, respectively.

To characterize the epitopes recognized by GW01, we determined the cryo-EM structure of S-6p^24^ bound to the GW01 Fab fragment at an overall resolution of 3.1 Å (Fig. 3a; Supplementary Figs. S4, S5 and Table S2). The resolution at the interface between RBD and GW01 was improved to 3.5 Å by applying focused refinement, allowing reliable model building.

S-6p bound to GW01 exhibits a conformation with two “up” RBDs (two RBD monomers are shown in orange and light blue) and one “down” RBD (gray), among which the two “up” RBDs are bound by GW01, whereas the “down” RBD is not (Fig. 3a). GW01 targets the lateral side of the RBD, which is highly conserved between SARS-CoV-2 and SARS-CoV. When aligned with the ACE2-binding site on RBD, we found only one overlapped residue, Gly502, between ACE2 and GW01. However, this was sufficient to allow competition between GW01 and ACE2 for binding to the RBD of the SARS-CoV-2 S protein (Fig. 3b, c).

Both the heavy chain and light chain of GW01 (GW01-H and GW01-L) participate in binding to the “up” RBD. The interface between RBD and GW01 consists mainly of a hydrophilic interaction network and can be divided into three clusters (Fig. 3d). RBD-Lys378 represents an important docking site that can form salt bridges with Asp106 of GW01-H and Glu111 of GW01-L and forms an H-bond with Asn109 of GW01-H. RBD-Tyr369 can also form hydrogen bonds with Asn52 and Lys67 of GW01-L and might be stabilized by Tyr110 of 4A8-H and Phe377 of RBD via π–π interactions. Furthermore, the main chain of Val503 and Gly504 of the RBD can be stabilized by Asp31 and Tyr102 of GW01-H through H-bonds.

We mapped the key residues that affected the neutralizing activities of GW01 by site-directed mutagenesis and found that the F374A, K378A, R408I, P463A, and G504N mutants greatly decreased the neutralization activities of GW01 (Fig. 3e). Residues F374, K378, R408, P463, and G504 were 99.99% conserved in a total of 1279,804 SARS-CoV-2 variant sequences (Fig. 3f; Supplementary Table S3). Residues F374, R408, and P463 were 100% conserved, and residues K378 and G504 were 86 and 48% conserved in different sarbecoviruses, respectively (Fig. 3g; Supplementary Table S4). Therefore, GW01 targets a conserved epitope shared by many different sarbecoviruses, resulting in extraordinary breadth for this NAb.

The epitopes of the reported cross-reactive NAbs S2A4, S304, and CR3022 (Fig. 3h) showed partial overlap with GW01 (Fig. 3i). However, GW01 binds to epitopes in the RBD of a wider range of sarbecoviruses than S2A4, S304, and CR3022. Consequently, GW01 is expected to be effective against variants with resistance to these NAbs. S309 binds to RBD in a different direction from GW01 (Fig. 3j), confirming different binding patterns in S309 and GW01.

### Identification of currently circulating SARS2-CoV-2 spike variants with resistance to the sarbecovirus NAbs and/or SARS-CoV-2 NAbs

**Fig. 4:**
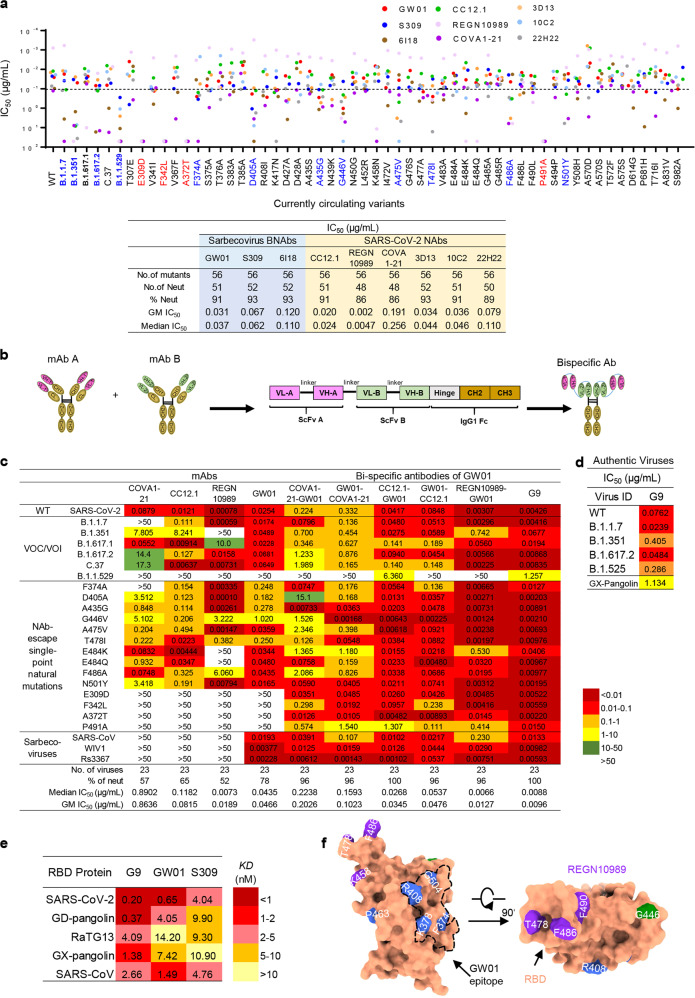
Neutralization of GW01-related bispecific antibodies against the currently circulating SARS-CoV-2 NAb-escape variants and sarbecoviruses. **a** Neutralization potency and breadth of mAbs against 56 currently circulating variants. Identification of putative NAb-escape variants by screening 56 currently circulating SARS2-CoV-2 variants, including B.1.1.7, B.1.351, and B.1.617.2, with sarbecovirus NAbs GW01, 6I18, and S309, as well as SARS-CoV-2 NAbs 3D13, 10C2, 22H22, CC12.1, and COVA1-21. **b** Bispecific antibody sequence and structure diagram. **c** Neutralization potency and breadth of GW01-related bispecific antibodies against a panel of 20 NAb-resistant variants, SARS-CoV and 2 bat SARSr-CoVs (WIV1 and Rs3367). **d** Neutralization of G9 against authentic SARS-CoV-2 and its variants B.1.1.7, B.1.351, B.1.617.2, and B.1.525, and authentic GX-pangolin. **e** Binding affinities of G9, GW01, and S309 binding to RBD proteins of five sarbecoviruses. **f** Neutralizing mechanism of bispecific antibody G9. Key mutated residues that can affect the neutralizing activity of the antibodies are mapped on the RBD. The purple block is for REGN10989, the blue block is for GW01, and the green block is for both REGN10989 and GW01. The black dotted line indicates the epitope of GW01 on RBD (orange). The epitope of REGN10989 is based on the results of hydrogen-deuterium exchange mass spectrometry.

It has been reported that naturally occurring spike VOCs, such as B.1.1.7, B.1.351, B.1.617.2, B.1.525, and B.1.1.529, have reduced sensitivity to SARS-CoV-2 NAbs^7^. To identify more NAb-escape variants, we constructed a panel of 56 pseudotyped SARS2-CoV-2 spike variants, including four VOCs (B.1.1.7, B.1.351, B.1.617.2, and B.1.1.529) and two VOIs (B.1.525, C.37), and 50 currently circulating variants with single-point mutations in the SARS-CoV-2 S protein according to the sequences from the GISAID and CNCB-NGDC databases^25,26^. We screened the variants with the sarbecovirus NAbs GW01, S309, and 6I18, as well as SARS-CoV-2 NAbs CC12.1^11^, REGN10989^18^, COVA1-21^13^, and 3D13, 10C2, 22H22 in a pseudotyped neutralization assay. GW01 neutralized 51 (91%) of the pseudoviruses in the panel with a median IC_50_ of 0.031 μg/mL, while S309 and 6I18 neutralized 52 (93%) of the pseudoviruses with median IC_50_ values of 0.067 μg/mL and 0.120 μg/mL, respectively. SARS-CoV-2 NAbs CC12.1, REGN10989, COVA 1-21, 3D13, 10C2, and 22H22 neutralized 51 (91%), 48 (86%), 48 (86%), 52 (93%), 51 (91%), and 50 (89%) of the pseudoviruses with GM IC_50_s of 0.020, 0.002, 0.191, 0.034, 0.036, and 0.079 μg/mL, respectively (Fig. 4a). Four mutants, E309D, F342L, A372T, and P491A, abolished sensitivity to both sarbecovirus NAbs and SARS-CoV-2 NAbs (Fig. 4a, red color). Moreover, eight variants, F374A, D405A, A435G, G446V, A475V, T478I, F486A, and N501Y, exhibited significantly reduced sensitivity to the tested antibodies (Fig. 4a, blue color). Therefore, in addition to B.1.1.7, B.1.351, B.1.617.2, and B.1.1.529, a number of currently circulating SARS-CoV-2 variants can escape neutralization by both sarbecovirus NAbs and SARS-CoV-2 NAbs.

### Neutralization of the bispecific NAb G9 against 23 currently circulating SARS2-CoV-2 NAb-escape variants and sarbecoviruses

We further evaluated the strategy of developing GW01 bispecific antibodies targeting different epitopes in RBD to increase their breadth and potency against the NAb-escape variants. We constructed six bispecific antibodies using GW01 in combination with the SARS-CoV-2 RBD-specific potent antibody REGN10989 or CC12.1 or the NTD-specific antibody COVA1-21. A bispecific antibody is a single gene-encoded IgG-like molecule consisting of single-chain variable fragments (scFvs) of the two parental neutralization antibodies, as well as the hinge, CH2, and CH3 regions of the IgG1 Fc domain^27^ (Fig. 4b). The sodium dodecyl sulfate-polyacrylamide gel electrophoresis (SDS-PAGE) results showed that the size of the six bispecific antibodies was ~70–80 kDa and that the purity was > 95% (Supplementary Fig. S6a). Cross-linking the bispecific antibodies with glutaraldehyde before SDS-PAGE showed a single band with a molecular weight of ~140–160 kDa (Supplementary Fig. S6b).

We measured the neutralizing activities of these six bispecific antibodies against 23 currently circulating NAb-escape variants, including SARS-CoV and SARSr-CoVs (WIV1 and Rs3367). All six GW01-related bispecific antibodies showed increased breadth and potency compared with their parental antibodies (Fig. 4c). Among these bispecific NAbs, GW01-REGN10989 (G9) was the broadest and most potent NAb, effectively neutralizing 100% of the NAb-escape variants, including B.1.1.529 and sarbecoviruses (23 out of 23) tested with a geometric mean (GM) IC_50_ of 8.8 ng/mL, while REGN10989 neutralized only 52% of 23 NAb-escape variants with a GM IC_50_ of 19 ng/mL (Fig. 4c). Moreover, G9 strongly neutralized authentic SARS-CoV-2 and its variants B.1.1.7, B.1.351, B.1.617.2, and B.1.525 with a median IC_50_ of 0.0762 μg/mL (Fig. 4d). G9 even neutralized authentic GX-pangolin coronavirus (Fig. 4d), which has 83.39% nucleotide similarity and 92.30% amino-acid similarity (Supplementary Fig. S7). G9 showed stronger binding affinities to the RBD proteins of SARS-CoV-2, RaTG13, GD-pangolin, GX-pangolin, and SARS-CoV than GW01 or S309 alone. GW01 exhibited higher binding affinities to most of the RBD proteins compared to S309, except the RBD of RaTG13 (Fig. 4e). These data suggested that GW01-related bispecific antibodies strongly enhanced neutralization breadth and potency against SARS-CoV-2 variants and other sarbecoviruses by increasing binding affinities to the RBD proteins.

### The neutralizing mechanism of the bispecific antibody G9

The high-resolution structure of the S-6p and GW01 complex provides an important clue to investigating the neutralizing mechanism of the bispecific antibody G9. Moreover, we mapped the key residues that affected the neutralizing activities of GW01 and REGN10989 by both alanine scanning and currently circulating mutations. Aligning the epitopes on the RBD of SARS-CoV-2 reveals clearly that GW01 and REGN10989 bind to different epitopes on the RBD from different directions (Fig. 4f; Supplementary Table S5). We thus propose that the bispecific antibody G9 can target the epitopes in RBD for both GW01 and REGN10989, consequently broadening the spectrum of its cross-neutralizing activity against the NAb-escape variants.

### Prophylactic and therapeutic administration of GW01 and G9 protected hACE2-transgenic mice from SARS-CoV-2-associated disease

**Fig. 5:**
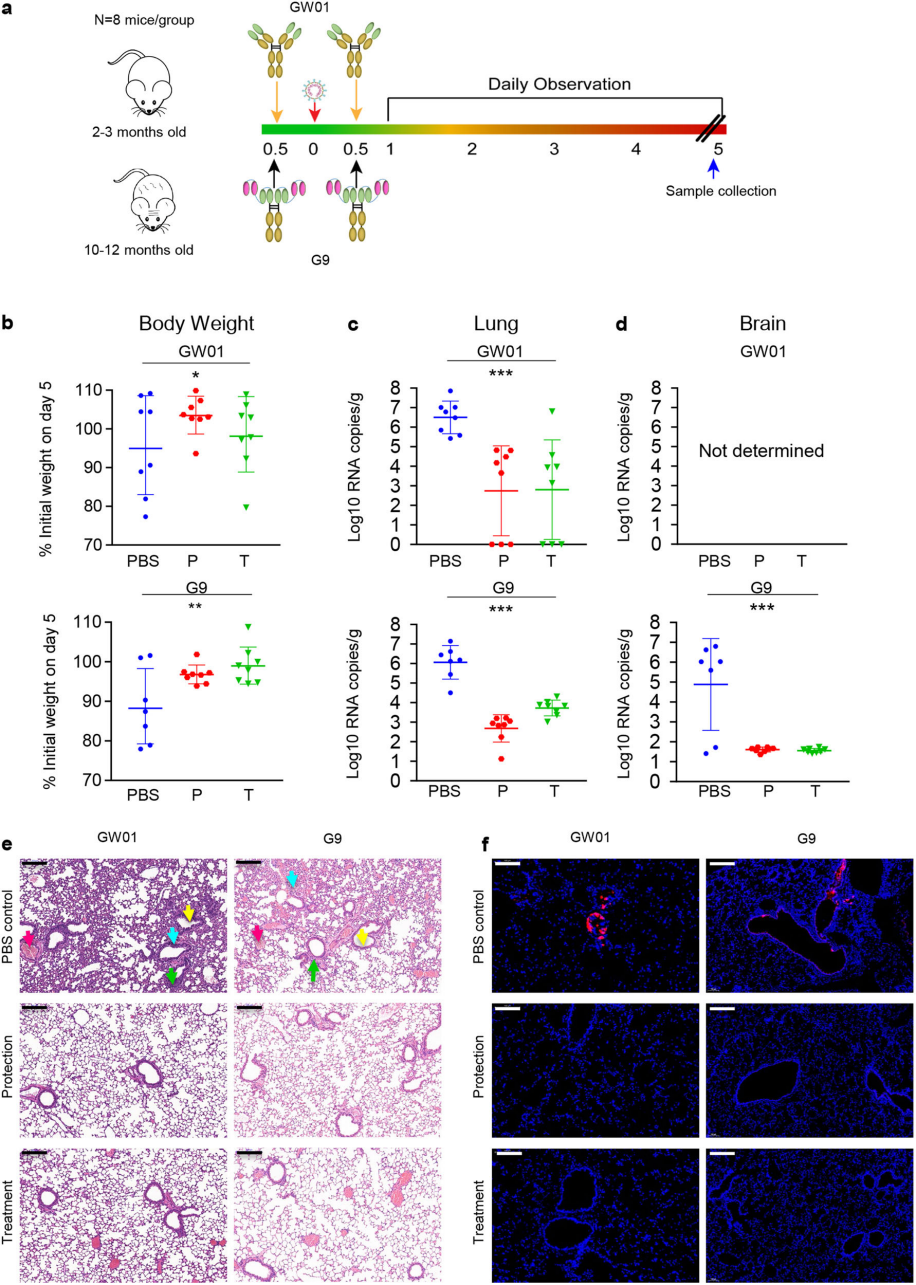
Prophylactic and therapeutic effect of G9 against SARS-CoV-2 infection of lung, brain, and associated disease in hACE2-transgenic mice. **a** Experimental scheme of the animal model. **b** Bodyweight changes in SARS-CoV-2-infected HFH4-hACE2-transgenic mice in the prevention group (P), treatment group (T), and PBS group of GW01 and G9. The percentage of weight change was calculated on day 5 for all animals. The symbol “+” signifies that the mice died. **c**, **d** Viral load as assessed by S gene RNA RT–PCR from lung tissue of the GW01 and G9 groups (**c**) or brain tissue from the G9 groups (**d**) on day 5 post infection. **e** Pathological changes in HFH4-hACE2-transgenic mouse lung on day 5 after SARS-CoV-2 infection. Mouse lung tissue was stained with H&E to observe pathological changes. Scale bars, 200 μm. **f** Viral antigen was detected by anti-SARS-CoV-2 N protein polyclonal antibody (red) in lung bronchi and alveoli collected 5 DPI. Images were collected using a Pannoramic MIDI system (3DHISTECH, Budapest). Scale bars, 100 μm.

The in vivo prophylactic and therapeutic potency of mAb GW01 and bispecific antibody G9 against SARS-CoV-2 was evaluated in hACE2-transgenic mice as previously described^28^. In the GW01 groups, 2- to 3-month-old mice were intraperitoneally injected with 200 μg of GW01 per mouse 12 h before or after the challenge with 5 × 10^5^ TCID_50_ SARS-CoV-2^4^. In the G9 groups, 10- to 12-month-old mice were intraperitoneally injected with 200 μg of antibody per mouse 12 h before or after challenge with 1 × 10^5^ TCID_50_ SARS-CoV-2 (Fig. 5a). Mice injected with phosphate-buffered saline (PBS) were challenged with the same dose of SARS-CoV-2 as controls. The bodyweight and survival rate of the mice were monitored for 5 days after the challenge. The protection group and treatment group of GW01 (Fig. 5b top) and G9 (Fig. 5b bottom) showed significant improvement in bodyweight over that of the PBS group (*P* < 0.05). We compared viral RNA copies in the lung tissue of the mice. Mice in the PBS group had significantly higher viral RNA copies (*P* < 0.0001) in the lung than the protection and treatment groups of mAb GW01 (Fig. 5c, top) or G9 (Fig. 5c, bottom). We also compared viral RNA copies in the brain tissue of the mice from the G9 groups. Mice in the protection and treatment groups of G9 had significantly lower viral RNA copies in the brain than the PBS group (*P* < 0.0001, Fig. 5d). Histological analysis of mouse lung pathological changes in the PBS group (Fig. 5e) revealed a resemblance to the effects of severe COVID-19 in humans. These changes were defined by the increased number of inflammatory cells around the bronchi and blood vessels (blue arrow), the falling off of bronchial epithelial cells (yellow arrow), widened and thickened alveolar walls (green arrow), and increased fibrin exudate in the alveolar cavity (red arrow). Both the protection group and the treatment group of GW01 and G9 exhibited fewer pathological changes (Fig. 5e) and less viral antigen (Fig. 5f) than the PBS group. The treatment groups showed a slightly higher level of pathological changes than the protection groups but fewer pathological changes overall than the PBS control group (Fig. 5e). Overall, GW01 and G9 showed prophylactic and therapeutic efficacy in hACE2-transgenic mice against bodyweight loss and viral replication, and pathological changes in the lung caused by SARS-CoV-2 infection.

## Discussion

A large number of SARS-CoV-2-specific NAbs have been isolated since the beginning of the COVID-19 pandemic, but most of them have only limited neutralization breadth. Here, we report the identification of a sarbecovirus NAb, GW01, from a donor who recovered from COVID-19. GW01 is different from the previously reported well-defined sarbecovirus NAbs ADG-2. ADG-2 shows no binding to the RBD in the S protein of RaTG13^15^, while GW01 is strongly bound to the RBD protein of RaTG13 with high binding affinity (Fig. 1a, b). The epitope of ADG-2 contains the residues D405, G502, G504, and Y505^15^ with conservation rates of 48%, 43%, 38%, and 95%, respectively, in different sarbecoviruses (Supplementary Table S4). The epitope of GW01 contains the residues F374, K378, R408, P463, and G504 with conservation rates of 100%, 86%, 100%, 100%, and 48%, respectively (Supplementary Table S4). Therefore, the epitope of GW01 partially overlaps the epitope of ADG-2 containing the residue G504, but it is more conserved than ADG-2 in different sarbecoviruses.

Sarbecovirus NAbs are capable of neutralizing not only known VOCs of SARS-CoV-2 but also other sarbecoviruses. However, in our study, we found that sarbecovirus NAbs could escape from the currently circulating mutants of SARS-CoV-2. The bispecific antibody CoV-X2, consisting of two SARS-CoV-2-specific antibodies, neutralized only SARS-CoV-2 variants^19^. We designed and constructed, for the first time, bispecific antibodies with sarbecovirus NAb. The bispecific antibody G9 showed extraordinary breadth and potency against SARS-CoV-2 variants and SARS-CoV, as well as SARSr-CoVs from bats (WIV1, RS3367) and pangolins (GX-pangolin), with a GM IC_50_ of 8.8 ng/mL (Fig. 4c).

Mutations in SARS-CoV-2 VOCs occur at residues S371L, K417, L452, T478, E484, and N501 in the receptor-binding motif at the top of the RBD. It is difficult to predict which mutations will predominate next as SARS-CoV-2 continues to evolve and circulate in human populations. We constructed 56 currently circulating variants, including Alpha, Beta, Delta, Omicron, Kappa, and Lambda. Twenty of these SARS-CoV-2 variants escaped neutralization by SARS-CoV-2 NAbs (Fig. 4a, c). Interestingly, all these NAb-escape variants, as well as SARS-CoV and SARSr-CoVs from bats (WIV1 and Rs3367), were potently neutralized by G9 (Fig. 4c). These results suggest the necessity of using bispecific antibodies or antibody cocktails for the treatment and prevention of infection by SARS-CoV-2 NAb-escape variants and SARS-CoV and SARSr-CoVs that may cause future emerging and re-emerging sarbecovirus diseases.

GW01 and G9 provided both prophylactic and therapeutic protection against SARS-CoV-2 infection in hACE2-transgenic mice. Furthermore, cryo-EM structure, competition assays, and site-directed mutagenesis analyses have revealed that GW01 binds to a conserved epitope in the RBD shared by divergent sarbecoviruses. However, GW01 and REGN10989 target different neutralizing epitopes in the RBD since they showed no competition in binding to the RBD (Fig. 2a). Importantly, the bispecific NAb G9 is bound to the epitopes in the RBD for both GW01 and REGN10989, thus exhibiting broad-spectrum cross-neutralizing activity against NAb-escape variants, including those with resistance to GW01 or REGN10989 alone. Indeed, G9 potently neutralized all 23 escape viruses tested, while GW01 and REGN10989 were able to neutralize 18 and 12 of them, respectively (Fig. 4c). Taken together, the results from this study suggest that the bispecific NAb G9 is a promising therapeutic and prophylactic candidate with the potential for the prevention and treatment of infection by SARS-CoV-2 and its NAb-escape variants, SARS-CoV, and SARSr-CoVs from bats and pangolins, which may cause future outbreaks of emerging and re-emerging coronavirus diseases.

## Materials and methods

### Cell lines, proteins, viruses, and plasmids

The human primary embryonic kidney cell lines (HEK293T) (CRL-3216™) and Huh-7 were obtained from the American Type Culture Collection (ATCC). Huh-7 cells were cultured in Dulbecco’s Modified Eagle’s Medium (DMEM) with 10% fetal bovine serum (FBS). In all, 85,000 CD19^+^IgA^−^IgD^−^IgM^−^ primary B cells were sorted out from peripheral blood mononuclear cells (PBMC) of recovered patients of COVID-19 and expanded in vitro for 13 days in Iscove’s Modified Dulbecco’s Medium (IMDM) in the presence of irradiated 3T3-msCD40L feeder cells, IL-2, and IL-21, as previously described^11^. SARS-CoV-2 RBD protein was purchased from GenScript (Nanjing, China). SARS-CoV-2 S1 and S2 proteins, as well as SARS-CoV S1 and RBD proteins, were purchased from Sino Biological (Beijing, China). RBD genes of RaTG13 (spike aa 330–583), pangolin-CoV-GD (spike aa 326–579), and pangolin-CoV-GX (spike aa 330–583) were synthesized (Sangon Biotech, Shanghai, China) and placed into an expression vector with an N-terminal signal peptide and an S-tag, as described previously^29^. The expression plasmids for SARS-CoV S protein, pcDNA3.1-SARS-CoV-S (GenBank accession: ABD72979.1), SARS-CoV-2 S protein, pcDNA3.1-SARS-CoV-2-S (GenBank accession: NC_045512)^4^, pcDNA3.1-WIV1 (GenBank accession: KC881007.1) and pcDNA3.1-Rs3367 (GenBank accession: KC881006.1) were synthesized by Genscript. The HIV-1 Env-deficient luciferase reporter vector pNL4-3. Luc. R-E- and 3T3mCD40L cells were obtained through the NIH AIDS Reagent Program. Authentic SARS-CoV-2 variants B.1.1.7, B.1.351, B.1.617.2, and B.1.525 were from Prof. Jincun Zhao (National Clinical Research Center for Respiratory Disease, Guangzhou Institute of Respiratory Health).

### Production of Pseudoviruses

S genes of SARS-CoV-2^30^, B.1.1.7 (69–70del, 144del, N501Y, A570D, D614G, P681H, T716I, S982A, D1118H), B.1.351 (D80A, D215G, 241–243del, K417N, E484K, N501Y, D614G, A701V), B.1.617.1 (L452R, E484K, D614G, P681R, Q1071H), B.1.617.2 (T19R, 157-158del, L452R, T478K, D614G, P681R, D950N), C.37 variant (G75V, T76I, 247–253del, L452Q, F490S, D614G, T859N), B.1.1.529 (A67V, 69–70del, T95I, G142D, 143-145del, N211I, 212del, ins215EPE, G339D, S371L, S373P, S375F, K417N, N440K, G446S, S477N, T478K, E484A, Q493R, G496S, Q498R, N501Y, Y505H, T547K, D614G, H655Y, N679K, P681H, N764K, D796Y, N856K, Q954H, N969K, L981F), SARS-CoV, bat SARSr-CoVs (WIV1 and Rs3367), GX-pangolin, and GD-pangolin were synthesized by BGI and constructed in pcDNA3.1 vector. Pseudoviruses were generated by cotransfection of 293 T cells with pNL4-3.Luc.R-E- backbone and viral envelope protein expression plasmids pcDNA3.1-SARS-CoV-2-S, pcDNA3.1-SARS-CoV-S, pcDNA3.1-BtSL-WIV1-S, or pcDNA3.1-BtSL-Rs3367. Fifty additional spike variants carrying currently circulating single-point mutations were constructed by site-directed mutagenesis.

### Neutralization assay

Neutralization activity of plasma from COVID-19 patients was measured using a single-round pseudovirus infection of Huh-7 cells^31^. Pseudoviruses could infect the same cells as those infected by SARS-CoV-2 or SARS-CoV. The neutralization assay was performed in accordance with the following steps. First, Huh-7 cells were seeded in a 96-well plate at a concentration of 10^4^ cells per well and cultured for 12 h. Then, 10 μL of mAb were 5-fold serially diluted with DMEM with 10% FBS and mixed with 40 μL of pseudovirus. The mixture was added to cultured Huh-7 for infection. The culture medium was refreshed after 12 h and incubated for an additional 48 h. Assays were developed with a luciferase assay system (Promega), and the relative light units (RLU) were read on a luminometer (Perkin Elmer). The titers of NAbs were calculated as NT_50_ and expressed as the highest dilution of plasma which results in a 50% reduction of luciferase luminescence compared with virus control.

### ELISA

RBD proteins of SARS-CoV-2, RaTG13 GX-pangolin, GD-pangolin, and SARS-CoV (1 μg/mL) were coated on a MaxiSorp Nunc-immuno 96-well plate (Thermo Scientific, USA) overnight at 4 °C. Wells were blocked with 5% non-fat milk (Biofroxx, Germany) in PBS for 1 h at room temperature, followed by incubation with serially diluted heat-inactivated sera or mAb in disruption buffer (PBS, 5% FBS, 2% bovine serum albumin (BSA), and 1% Tween-20) for 1 h at room temperature. A 1:2500 dilution of horseradish peroxidase-conjugated goat anti-human IgG antibody (Jackson Immuno Research Laboratories, USA) was added for 1 h at room temperature. Wells were washed five times between each step with 0.2% Tween-20 in PBS. Wells were developed using ABST (Thermo Scientific, USA) for 30 min and read at 405 nm on a Multiskan FC plate reader (Thermo Scientific, USA).

### Memory B-cell staining, sorting, and antibody cloning

SARS-CoV-2-specific monoclonal antibodies were isolated from PBMC of recovered patients by in vitro single B cells, as previously described^22^. In brief, 85,000 CD19^+^IgA^−^IgD^−^IgM^−^ memory B cells were sorted and resuspended in a medium with IL-2, IL-21, and irradiated 3T3-msCD40L feeder cells, followed by seeding into a 384-well plate at a density of four cells per well. After 13 days of incubation, supernatants from each well were screened for neutralization activity using a high-throughput microneutralization assay against SARS-CoV-2 and SARS-CoV. From the wells with positive scores in the neutralization assay, the variable region of the heavy chain and the light chain of the immunoglobulin gene was amplified by reverse transcription-polymerase chain reaction (RT–PCR) and re-expressed as described previously^32^. The full-length IgG was purified using a protein G column (Smart-Lifesciences).

### Biolayer interferometry (BLI) binding assay

The kinetics of monoclonal antibody binding to RBD proteins was measured by BLI binding assay on a FortéBio OctetRED96 instrument, using anti-human IgG (AHC) biosensors, as previously described. The assay followed sequential steps at 30 °C as follows. (1) Baseline: biosensors immersed in sterile water for 60 s. (2) mAb loading: biosensors immersed 200 s with mAb at 10 μg/mL. (3) Wash: biosensors immersed with 0.02% PBST (PBS with 0.02% Tween) for 120 s to reach baseline. (4) Association: biosensors immersed with serial diluted SARS-CoV-2 RBD or SARS-CoV RBD at 6 μg/mL for 300 s. (5) Dissociation: biosensors immersed with 0.02% PBST for 300 s to reach baseline. The buffer control binding was subtracted to deduct nonspecific binding. *K*_on_, *K*_off_, and *K*D were calculated by FortéBio Data Analysis software (Version 8.1), using 1:1 binding and a global fitting model.

### Biolayer interferometry competition assay

Ab cross-competition was conducted following the classical sandwich assay. (1) Baseline: biosensors immersed in sterile water for 60 s. (2) 1^st^ mAb loading: biosensors immersed 200 s with mAb1 at 10 μg/mL. (3) Wash: biosensors immersed with 0.02% PBST for 120 s to reach baseline. (4) Blocking: biosensors immersed in IgG1 isotype control at 50 μg/mL for 200 s. (5) Association: biosensors immersed with SARS-CoV-2 RBD or SARS-CoV RBD at 6 μg/mL for 300 s. (6) Wash: biosensors immersed with 0.02% PBST for 120 s to reach baseline. (7) Competition: biosensors immersed with mAb2 at 10 μg/mL for 600 s to detect the association between mAb2 and SARS-CoV-2 RBD or SARS-CoV RBD.

For ACE2 competition, biosensors were immersed with 20 μg/mL of ACE2-Fc for 600 s. After baseline, wash, and blocking steps, biosensors were immersed with pre-mix of 600 nM of mAb and 100 nM SARS-CoV-2 RBD or SARS-CoV RBD for 600 s. A mixture of ACE2-Fc and SARS-CoV-2 RBD or SARS-CoV RBD was used as a positive control, while the mixture of IgG1 isotype control and SARS-CoV-2 RBD or SARS-CoV RBD was used as a negative control.

### Plaque reduction assay

The inhibition of antibodies against live authentic SARS-CoV-2, bat SARSr-CoV (WIV1 and SHC014), and SARS-CoV-2 variant B.1.351 was performed in the biosafety level 3 facility (BSL3) at Wuhan Institute of Virology. The neutralization of antibodies on the coronaviruses was determined by plaque reduction assay on Vero-E6 cells, as previously described^28^. Briefly, serially diluted antibodies were incubated with 100 plaque-forming units of viruses at 37 °C for 30 min. The mixtures were added to the monolayer of Vero-E6 cells. After adsorption at 37 °C for 1 h, the supernatant was removed, and a 0.9% methylcellulose overlay was added. After 3-day culture, plaques were developed and counted by fixing with 4% formaldehyde and staining with 0.5% crystal violet.

### Construction and expression of sarbecovirus bispecific NAbs

Genes of a bispecific Ab consisting of the scFv of GW01 and scFv of COVA1-21, CC12.1, or REGN10989 were synthesized and codon-optimized by GenScript. The bispecific antibody sequence alignment was as follows: variable light chain (VL) and variable heavy chain (VH) of mAb A or mAb B were linked with a (Gly_4_Ser)_3_ linker. VL-VH of mAb A and VL-VH of mAb B were linked with a GlySer(Gly_4_Ser)_4_ linker and then fused to the expression vector with hinge-CH2-CH3 fragment of human immunoglobulin (hIgG1 Fc). G9 bispecific antibody sequence order was as follows: GW01 VL-(Gly_4_Ser)_3_-GW01 VH-GlySer(Gly_4_Ser)_4_-REGN10989 VL-(Gly_4_Ser)_3_-REGN10989 VH-hinge-CH2-CH3.

For expression of bispecific Abs, HEK 293 F cells were transiently transfected with plasmid encoding constructed bispecific Abs genes. After culturing for 6 days at 37 °C in a 5% CO_2_ incubator, Ab-containing culture supernatants were harvested using protein G beads (Smart-Lifesciences), according to the manufacturer’s protocol. Purified bispecific antibodies were concentrated by using ultra centrifugal filters (50 kD; Millipore) and stored in PBS at −80 °C.

### SDS-PAGE and cross-linking SDS-PAGE of bispecific antibodies

The purity and molecular weight of bispecific antibodies were then analyzed by SDS-PAGE and cross-linking SDS-PAGE. In brief, 5 μg of bispecific antibodies were mixed with 5× SDS-loading sample buffer containing 10% β-mercaptoethanol. The samples were heated for 10 min at 100 °C and were then loaded on an SDS gradient gel (4%–20% Precast Protein Improve Gels, Genscript Biotech Corporation). The gel was run for 120 min at 120 V, and Coomassie staining was performed.

The extent of the dimer was investigated by cross-linking of bispecific antibodies with glutaraldehyde (Sigma-Aldrich). In brief, 5 μg of antibodies were diluted in 25 μL of PBS in the presence of a 2.7 µM of glutaraldehyde cross-linker. The mixture was incubated at RT for 5 min, and then glutaraldehyde was quenched by adding 1 M Tris-HCl buffer (pH 8.0) to a final concentration of 40 mM. After mixing with 5× SDS-loading sample, the protein samples were loaded on a 4%–20% SDS gradient gel. The gel was run for 180 min at 120 V and confirmed by Coomassie staining.

### Prophylactic and therapeutic mouse model

To test the prophylactic and therapeutic effects of GW01, 2–3-month-old mice (four males and four females) were intraperitoneally injected with 200 μg/mouse of mAb GW01 12 h before or after challenge with 5 × 10^5^ TCID_50_ SARS-CoV-2. When we tested the prophylactic and therapeutic effects of bispecific antibody G9, only 10–12-month-old mice were available. We lowered the challenge titer of SARS-CoV-2 to 1 × 10^5^ TCID_50._ Each mouse was intraperitoneally injected with 200 μg/mouse of G9 12 h after challenge with 1 × 10^5^ TCID_50_ SARS-CoV-2. Mice injected with PBS were used as controls. The number of each group of mice was 8, except for the PBS group of G9, which was 7, since we had a limited number of 10–12-month-old mice. Mouse bodyweight was monitored as an indicator of disease progression. On day 5, lung tissue samples were collected for viral burden assessment. Viral infections were performed in a BSL3 facility in accordance with recommendations for the care and use of laboratory animals and the Institutional Review Board of the Wuhan Institute of Virology, CAS (Ethics Number WIVA05202017).

### Extraction of viral RNA and qRT–PCR

Mouse organs were homogenized in DMEM, and viral RNA was isolated using the QIAamp ® Viral RNA Mini Kit (QIAGEN). HiSxript ®II One step qRT–PCR SYBR® Green Kit (Vazyme) was used to amplify the selected genes by real-time quantitative PCR, and 2 μL of RNA were used as a template. Viral genomic copies were calculated by average values from duplicates of each gene. The primers were designed based on the SARS-CoV-2 S gene as follows: RBD-qF1: 5′-CAATGGTTTAACAGGCACAGG-3′; RBD-qR1: 5′-CTCAAGTGTCTGTGGATCACG-3′. The PCR system followed the protocol of the previous study using a Step-One Plus Real-time PCR machine (ABI)^3^.

### Histological analysis

Pathology was performed on mice sacrificed on day 5 post infection. The lung samples were fixed with 4% paraformaldehyde, embedded in paraffin, and cut into 3.5 μm sections. The fixed tissue samples were stained with hematoxylin–eosin (H&E), and the SARS-CoV-2 antigen was detected by indirect immunofluorescence (IFA). The tissue sections were stained with H&E for routine histology. For IFA, the slides were dewaxed and rehydrated, followed by thermally induced antigen repair within 15 min in the microwave with ethylenediamine tetraacetic acid. The slides were washed with PBS and 0.02% Triton X-100 and then sealed with 5% BSA for 1 h at room temperature. Primary antibody (rabbit anti-SARS-CoV-2 N protein polyclonal antibody, made in-house: 1:500) was dropped onto the slides and then washed in PBS. When the slide was nearly dry, the tissue was covered with Cy3-conjugated goat anti-rabbit IgG (ABCAM, AB6939) at a 1:200 dilution. After washing with PBS, the slides were stained with DAPI (Beyotime) at a 1:100 dilution. The image information was collected using a Pannoramic MIDI system (3DHISTECH, Budapest).

### Protein expression and purification

The S-6p construct^24^ of SARS-CoV-2 S protein (GenBank ID: QHD43416.1) was cloned into the pCAG vector (Invitrogen) with six proline substitutions at residues 817, 892, 899, 942, 986 and 987, a “GlySerAlaSer” substitution at residues 682 to 685 and a C-terminal T4 fibritin trimerization motif followed by one Flag tag. The mutants were generated with a standard two-step PCR-based strategy.

The recombinant S-6p protein was overexpressed using HEK 293 F mammalian cells (Invitrogen) cultured in SMM 293T-II medium (Sino Biological, Inc.) at 37 °C under 5% CO_2_ in a Multitron-Pro shaker (Infors, 130 rpm). When the cell density reached ~2.0 × 10^6^ cells/mL, the plasmid was transiently transfected into the cells. For one liter of cell culture, about 1.5 mg of the plasmid were premixed with 3 mg of polyethylenimines (Polysciences) in 50 mL of fresh medium for 15 min before adding to cell culture. The supernatant was collected by centrifugation at 3800× *g* for 12 min after 60 h of transfection. The secreted extracellular domain of spike protein (S-ECD) proteins were purified by anti-FLAG M2 affinity resin (Sigma-Aldrich). After loading two times, the anti-FLAG M2 resin was washed with the wash buffer containing 25 mM Tris (pH 8.0) and 150 mM NaCl. The protein was eluted with the wash buffer plus 0.2 mg/mL flag peptide. The eluent was then concentrated and subjected to size-exclusion chromatography (Superose 6 Increase 10/300 GL, GE Healthcare) in buffer containing 25 mM Tris (pH 8.0) and 150 mM NaCl. The peak fractions were collected and concentrated to incubate with Fab-GW01. Purified S-6p was mixed with Fab-GW01 at a molar ratio of about 1:3.6 for 1 h. Then the mixture was subjected to size-exclusion chromatography (Superose 6 Increase 10/300 GL, GE Healthcare) in buffer containing 25 mM Tris (pH 8.0) and 150 mM NaCl to remove excess Fab-GW01. The peak fractions were collected for EM analysis.

### Cryo-EM sample preparation

The peak fractions of complex were concentrated to about 1.5 mg/mL and applied to the grids. Aliquots (3.3 μL) of the protein complex were placed on glow-discharged holey carbon grids (Quantifoil Au R1.2/1.3). The grids were blotted for 2.5 s or 3.0 s and flash-frozen in liquid ethane cooled by liquid nitrogen with Vitrobot (Mark IV, Thermo Scientific). The prepared grids were transferred to a Titan Krios operating at 300 kV equipped with Gatan K3 detector and GIF Quantum energy filter. Movie stacks were automatically collected using AutoEMation^33^ with a slit width of 20 eV on the energy filter and a defocus range from –1.2 µm to –2.2 µm in super-resolution mode at a nominal magnification of ×81,000. Each stack was exposed for 2.56 s with an exposure time of 0.08 s per frame, resulting in a total of 32 frames per stack. The total dose rate was ~50 e^−^/Å^2^ for each stack. The stacks were motion-corrected with MotionCor2^34^ and binned 2-fold, resulting in a pixel size of 1.087 Å/pixel. Meanwhile, dose weighting was performed^35^. The defocus values were estimated with Gctf^36^.

### Data processing

Particles for S-ECD in complex with GW01 were automatically picked using Relion 3.0.6^37–40^ from manually selected micrographs. After 2D classification with Relion, good particles were selected and subjected to two cycles of heterogeneous refinement without symmetry using cryoSPARC^41^.The good particles were selected and subjected to non-uniform refinement (beta) with C1 symmetry, resulting in 3D reconstruction for the whole structures and then further subjected to 3D auto-refinement and post-processing with Relion. For interface between S protein of SARS-CoV-2 and GW01, the dataset was subjected to focused refinement with adapted mask on each RBD-GW01 subcomplex to improve map quality. Similar RBD-GW01 subcomplexes were combined into one data set, if possible and necessary. The re-extracted dataset was 3D-classified with Relion focused on RBD-GW01 subcomplex. Then the good particles were selected and subjected to focused refinement with Relion, resulting in 3D reconstruction of better quality on RBD-GW01 subcomplex.

The resolution was estimated with the gold-standard Fourier shell correlation 0.143 criterion^42^ with high-resolution noise substitution^43^. Refer to Supplementary Figs. S4, S5, and Supplementary Table S2, for details of data collection and processing.

### Model building and structure refinement

For the model building of the complex of SARS-CoV-2 S-ECD/GW01, the atomic model of S-ECD in complex 4A8 (PDB ID: 7C2L) was used as a template, which was molecular dynamics flexible fitted^44^ into the whole cryo-EM map of the complex and the focused refined cryo-EM map of the RBD-GW01 subcomplex, respectively. A Chainsaw^45^ model of GW01 was first obtained using 4A8 as a template, which was further manually adjusted based on the focused refined cryo-EM map of the RBD-GW01 subcomplex with Coot^46^. Each residue was manually checked with the chemical properties taken into consideration during model building. Several segments, the corresponding densities of which were invisible, were not modeled. Structural refinement was performed in Phenix^47^ with secondary structure and geometry restraints to prevent overfitting. To monitor the potential overfitting, the model was refined against one of the two independent half maps from the gold-standard 3D refinement approach. Then, the refined model was tested against the other map. Statistics associated with data collection, 3D reconstruction, and model building were summarized in Supplementary Table S2.

### Statistical analysis

Statistical analyses were carried out using GraphPad Prism 7.0. Significant differences between groups were determined using a one-way analysis of variance. Statistical significance: *P < 0.05, **P < 0.01, ***P < 0.001, ****P < 0.0001.

## Supplementary information

Supplementary Information

## Data Availability

Atomic coordinates and cryo-EM density maps of the complex of SARS-CoV-2 S-ECD/GW01 (PDB: 7EPX; whole map: EMD-31249, antibody-epitope interface-focused refined map: EMD-31250) have been deposited to the Protein Data Bank (http://www.rcsb.org) and the Electron Microscopy Data Bank (https://www.ebi.ac.uk/pdbe/emdb/), respectively. All data from this study are included within this manuscript/Supplementary Material and are available from the Lead Contact (Jinghe Huang, jinghehuang@fudan.edu.cn) upon request.
