# Supplementary material for: Novel sarbecovirus bispecific neutralizing antibodies with exceptional breadth and potency against currently circulating SARS-CoV-2 variants and sarbecoviruses
Source: Cell Discov. 2022 Apr 21;8:36. doi: 10.1038/s41421-022-00401-6 (PMC9021188; doi:10.1038/s41421-022-00401-6)
Supplement: Supplementary file 1 — Supplementary Information [file 41421_2022_401_MOESM1_ESM.pdf]

Supplemental Information for

Novel sarbecovirus bispecific neutralizing antibodies with exceptional breadth and potency against currently circulating SARS-CoV-2 variants and sarbecoviruses

Yingdan Wang[1#], Meiqin Liu[2,5#], Yaping Shen[1,3,6#], Yunping Ma[1,4#], Xiang Li[1#], Yuanyuan Zhang[3,6#], Mei Liu[1#], Xing-Lou Yang[2#], Jun Chen[1#], Renhong Yan[3,6], Die Luan[1], Yanqun Wang[7,8], Ying Chen[2,5], Qimin Wang[1], Haofeng Lin[2,5], Yaning Li[9], Kaiyue Wu[1], Tongyu Zhu[1], Jincun Zhao[7,8], Hongzhou Lu[1]*, Yumei Wen[1]*, Shibo Jiang[1]*, Fan Wu[4]*, Qiang Zhou[3,6]*, Zheng-Li Shi[2]*, Jinghe Huang[1]*

[1]Key Laboratory of Medical Molecular Virology (MOE/NHC/CAMS) and Shanghai Institute of Infectious Disease and Biosecurity, Shanghai Public Health Clinical Center, School of Basic Medical Sciences, Fudan University, Shanghai, China.
[2]CAS Key Laboratory of Special Pathogens, Wuhan Institute of Virology, Center for Biosafety Mega-Science, Chinese Academy of Sciences, Wuhan, Hubei, People's Republic of China.
[3]Center for Infectious Disease Research, Westlake Laboratory of Life Sciences and Biomedicine, Key Laboratory of Structural Biology of Zhejiang Province, School of Life Sciences, Westlake University, 18 Shilongshan Road, Hangzhou, Zhejiang Province, China.
[4]Shanghai Immune Therapy Institute, Shanghai Jiao Tong University School of Medicine Affiliated Renji Hospital, Shanghai 200127, China.
[5]University of Chinese Academy of Sciences, Beijing, People's Republic of China.
[6]Institute of Biology, Westlake Institute for Advanced Study, 18 Shilongshan Road, Hangzhou, Zhejiang Province, China.
[7]State Key Laboratory of Respiratory Disease, National Clinical Research Center for Respiratory Disease, Guangzhou Institute of Respiratory Health, the First Affiliated Hospital of Guangzhou Medical University, Guangzhou, Guangdong, China;
[8]Institute of Infectious Disease, Guangzhou Eighth People's Hospital of Guangzhou Medical University, Guangzhou, Guangdong, China.
[9]Beijing Advanced Innovation Center for Structural Biology, Tsinghua-Peking Joint Center for Life Sciences, School of Life Sciences, Tsinghua University, Beijing, China.

[#]These authors contributed equally.
*Correspondence to: Dr. Jinghe Huang, Email: Jinghehuang@fudan.edu.cn; Dr. Zheng-Li Shi, Email: zlshi@wh.iov.cn; Dr. Qiang Zhou, Email: zhouqiang@westlake.edu.cn; Dr. Fan Wu, Email: wufan@fudan.edu.cn; Dr. Shibo Jiang, Email: shibojiang@fudan.edu.cn; Dr. Yumei Wen, Email: ymwen@shmu.edu.cn; Dr. Hongzhou Lu, Email: luhongzhou@fudan.edu.cn.

Keywords: COVID-19; SARS-CoV-2; Sarbecovirus bispecific NAb; Variants

Table S1. The germline and CDRH3 sequences of GW01, 6I18, 3D13, 10C2, and 22H22.

Donor ID	mAb ID	VH (% identity)	CDRH3 sequence	VL (% identity)	CDRL3 sequence
Donor 1	GW01	IGHV3-43*02 (96.53)	AKDRSYGPPDVFNYEYGMDV	IGLV1-44*01 (100)	AAWDDSLNWV
Donor 1	6I18	IGHV4-59*01 (98.6)	ATDYYDSSGYSYGMDV	IGKV1-12*01 (98.92)	QQANNFPRT
Donor 2	3D13	IGHV3-53*01 (98.6)	ARDLSSAGGMDV	IGKV3-20*01 (99.65)	QQYGSSPGT
Donor 2	10C2	IGHV3-53*01 (99.3)	ARLLVATIRDFDY	IGKV3-15*01 (100)	QQYNNWPPWT
Donor 2	22H22	IGHV3-53*01 (98.95)	ARGYGDYENYFDY	IGKV1-9*01 (95.7)	QHLWT

Table S2. Data collection, 3D reconstruction and model statistic

**Data collection**		
EM equipment	Titan Krios (Thermo Fisher Scientific)	
Voltage (kV)	300	
Detector	Gatan K3 Summit	
Energy filter	Gatan GIF Quantum, 20 eV slit	
Pixel size (Å)	1.087	
Electron dose (e-/Å2)	50	
Defocus range (μm)	-1.2 ~ -2.2	
Number of collected micrographs	1,538	
Number of selected micrographs	1,483	
Sample	S-ECD in complex with GW01	
**3D reconstruction**		
	Whole model	Interface between RBD and GW01
Software	cryoSPARC/ Relion	Relion
Number of used particles	309,831	203,736
Resolution (Å)	3.0	3.5
Symmetry	C1	
Map sharpening B factor ($Å^2$)	-90	
**Refinement**		
Software	Phenix	
Cell dimensions (Å)	313.056	
Model composition		
Protein residues	3,862	
Side chains assigned	3,862	
Sugar	71	
R.m.s deviations		
Bond lengths (Å)	0.007	
Bond angles (˚)	0.985	
Ramachandran plot statistics (%)		
Preferred	91.84	
Allowed	7.99	
Outlier	0.17	

Table S3. Conservation rates of GW01 epitope in SARS-CoV-2 variants.

Total SARS-CoV-2 variation sequences number:		1279804		
F374				
mutation site	protein site	Total frequency	T. Mut. Freq.	T. Cons. Freq.
22682: 'T->C'	F374L	8	0.0009%	100.00%
22683: 'T->A'	F374Y	4		
K378				
mutation site	protein site	Total frequency	T. Mut. Freq.	T. Cons. Freq.
22694: 'A->C'	K378Q	1	0.0074%	99.99%
22694: 'A->G'	K378E	2		
22695: 'A->G'	K378R	16		
22695: 'A->T'	K378M	1		
22696: 'G->T'	K378N	75		
R408				
mutation site	protein site	Total frequency	T. Mut. Freq.	T. Cons. Freq.
22784: 'A->G'	R408G	41	0.0244%	99.98%
22785: 'G->A'	R408K	81		
22785: 'G->C'	R408T	6		
22785: 'G->T'	R408I	177		
22786: 'A->T'	R408S	7		
A435				
mutation site	protein site	Total frequency	T. Mut. Freq.	T. Cons. Freq.
22865: 'G->A'	A435T	12	0.0057%	99.99%
22865: 'G->T'	A435S	61		
P463				
mutation site	rotein site(actua	Total frequency	T. Mut. Freq.	T. Cons. Freq.
22949: 'C->G'	P463A	2	0.0080%	99.99%
22949: 'C->T'	P463S	99		
22950: 'C->A'	P463H	1		
22950: 'C->T'	P463L	1		

T. Mut. Freq. : Total mutation frequency

T. Cons. Freq.: Total conservation frequency

Sequences of SARS-CoV-2 variants are from https://weilab.math.msu.edu/MutationAnalyzer/

Table S4. Conservation rates of GW01 and ADG-2 epitopes in different sarbecoviruses

Conservation rates of GW01 epitope in different sarbecoviruses

Sarbecoviruses\ Residues	F374	K378	R408	P463	G504
SARS-CoV-2	F	K	R	P	G
SARS-CoV	F	K	R	P	G
Pangolin BetaCoV GD	F	K	R	P	G
Bat CoV RaTG13	F	K	R	P	G
Pangolin BetaCoV GX	F	K	R	P	G
SARS-CoV SZ3	F	K	R	P	G
SARS-CoV BJ01	F	K	R	P	G
Bat SARSr-CoV WIV1	F	K	R	P	G
Bat SARSr-CoV Rs3367	F	K	R	P	G
Bat SARSr-CoV WIV16	F	K	R	P	G
Bat SARSr-CoV ZXC21	F	K	R	P	E
Bat SARSr-CoV ZC45	F	K	R	P	E
Bat SARSr-CoV YNLF31C	F	N	R	P	E
Bat SARSr-CoV SX2013	F	N	R	P	E
Bat SARSr-CoV Rf1	F	N	R	P	E
Bat SARSr-CoV Rp3	F	K	R	P	A
Bat SARSr-CoV GX2013	F	K	R	P	A
Bat SARSr-CoV HKU3-1	F	K	R	P	A
Bat SARSr-CoV Longquan-140	F	K	R	P	A
Bat SARSr-CoV SC2018	F	K	R	P	A
Bat SARSr-CoV HuB2013	F	K	R	P	A
Conservation (%)	100	86	100	100	48

Conservation rates of ADG-2 epitope in different sarbecoviruses

Sarbecoviruses \ Residues	D405	G502	G504	Y505
SARS-CoV-2	D	G	G	Y
SARS-CoV	D	G	G	Y
Pangolin BetaCoV GD	D	G	G	Y
Bat CoV RaTG13	D	G	G	H
Pangolin BetaCoV GX	D	G	N	Y
SARS-CoV SZ3	D	G	G	Y
SARS-CoV BJ01	D	G	G	Y
Bat SARSr-CoV WIV1	D	G	G	Y
Bat SARSr-CoV Rs3367	D	G	G	Y
Bat SARSr-CoV WIV16	D	P	E	Y
Bat SARSr-CoV ZXC21	S	P	E	Y
Bat SARSr-CoV ZC45	S	P	E	Y
Bat SARSr-CoV YNLF31C	S	P	E	Y
Bat SARSr-CoV SX2013	S	P	E	Y
Bat SARSr-CoV Rf1	S	P	A	Y
Bat SARSr-CoV Rp3	S	P	A	Y
Bat SARSr-CoV GX2013	S	P	A	Y
Bat SARSr-CoV HKU3-1	S	P	A	Y
Bat SARSr-CoV Longquan-140	S	P	A	Y
Bat SARSr-CoV SC2018	S	P	A	Y
Bat SARSr-CoV HuB2013	S	P	A	Y
Conservation (%)	48	43	38	95

Table S5. Neutralization of GW01 and REGN10989 against a panel of 63 SARS-CoV-2 alanine mutants and naturally occurring mutants.

	IC_{50} Fold change	
Mutants	GW01	REGN10989
SARS2 WT	1	1
T307E	0	2
E309D	>1000	>10000
V341I	5	3
F342L	>1000	>10000
V367F	1	3
Y369A	3	6
A372T	>1000	>10000
F374A	10	4
S375A	3	1
T376A	>1000	>10000
F377A	>1000	>10000
K378A	>1731	2
S383A	2	1
T385A	0	2
K386A	>1000	>10000
F392A	8	3
D405A	7	0
R408I	11	3
Q414E	2	7
K417N	4	1
D427A	1	3
D428A	0	2
A435S	0	3
A435G	10	3
N439K	1	5
G446V	40	>10000
N450G	1	2
L452R	1	4
K458N	0	>10000
P463A	58	2
I472V	3	1
A475V	1	2
G476S	0	3
S477A	6	3
T478I	10	486
V483A	2	3
E484A	1	2
E484K	1	>10000
G485A	1	2
F486A	2	>10000
G485R	1	40
F486L	1	58
N487A	2	1
Y489A	>1000	>10000
F490L	1	639
P491A	>1000	>10000
S494P	1	37
N501Y	1	10
G504N	18	2
Y505A	>1000	>10000
Y508H	2	3
E516A	>1000	>10000
L517A	0	2
I569V	>1000	>10000
A570D	0	1
A570S	1	4
T572F	1	3
A575S	1	3
D614G	3	4
P681H	5	1
T716I	0	1
A831V	1	2
S982A	4	1

Residues that decreased the sensitivity (fold change >10) only of GW01 are highlighted in blue. Residues that decreased the sensitivity (fold change >100) only of REGN10989 are highlighted in purple.

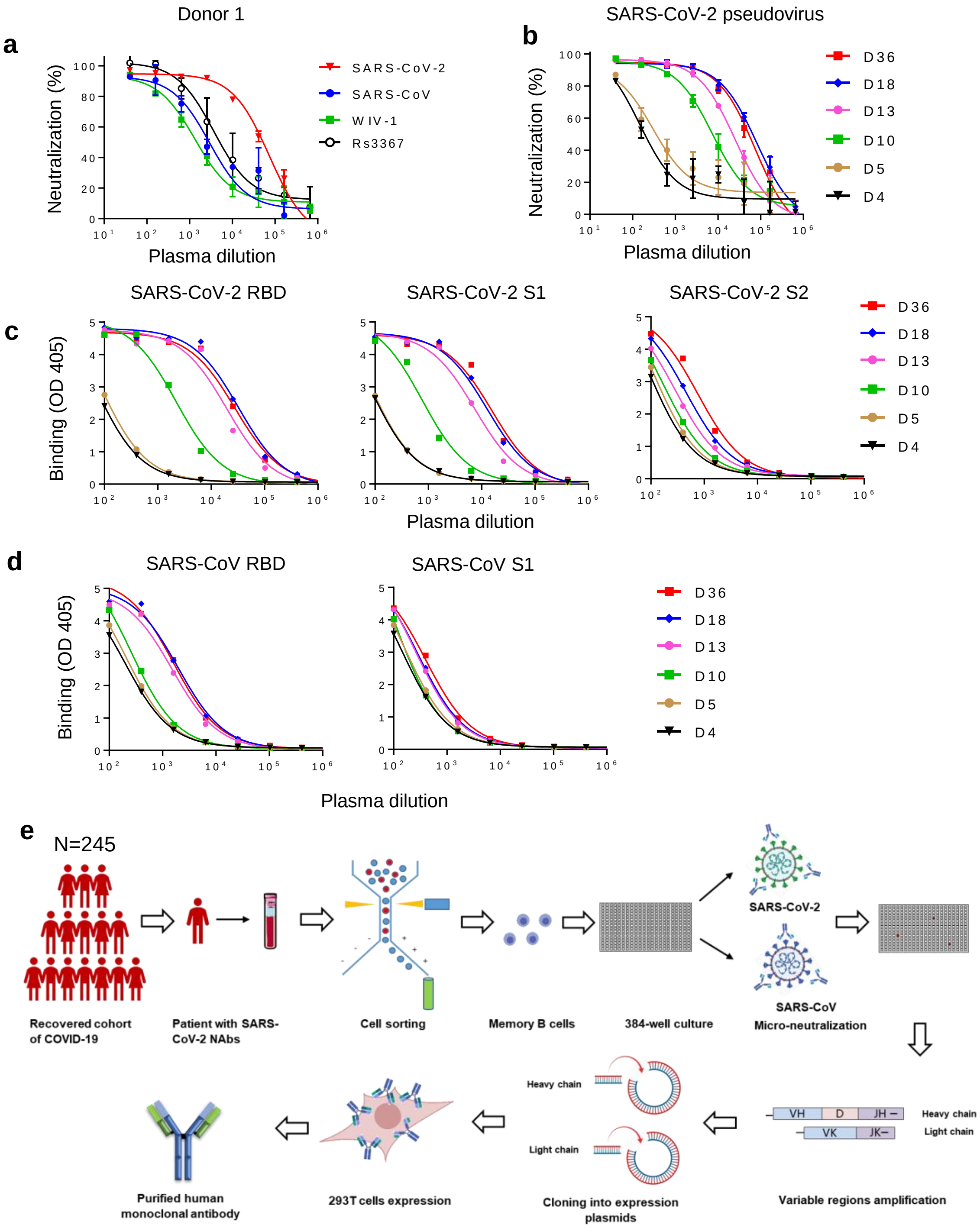

Fig. S1. Donor 1 recovered from COVID-19 with potent cross-neutralizing sera against SARS-CoV-2, SARS-CoV, Bat SARSr-CoV WIV1 and Rs3367.

(**a**) Neutralization of SARS-CoV-2, SARS-CoV, Bat SARSr-CoV WIV1 and Rs3367 by plasma collected from Donor 1. Sequential plasma samples collected from Donor 1 at different time points after admission were evaluated for SARS-CoV-2-specific NAb titres (**b**), SARS-CoV-2 RBD-, S1-, and S2-binding antibodies (**c**) and SARS-

CoV RBD- and S1-binding antibodies (**d**). (**e**) Pipeline to isolate NAbs from patients who recovered from COVID-19.

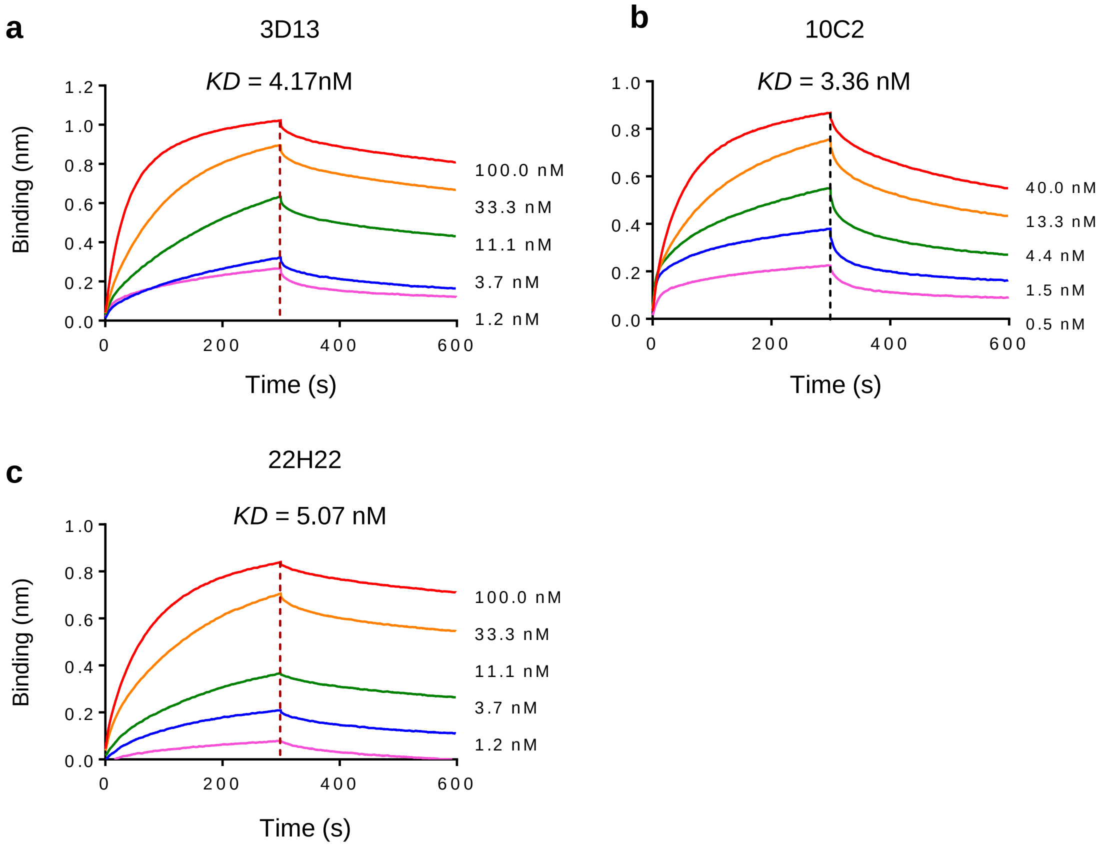

Fig. S2. The binding affinity of 3D13, 10C2, and 22H22 to RBD SARS-CoV-2 was measured by bilayer interferometry experiments.

3D13 (**a**), 10C2 (**b**), and 22H22 (**c**) were immobilized on anti-human IgG (AHC) biosensors and then tested for their binding ability to SARS-CoV-2 RBD.

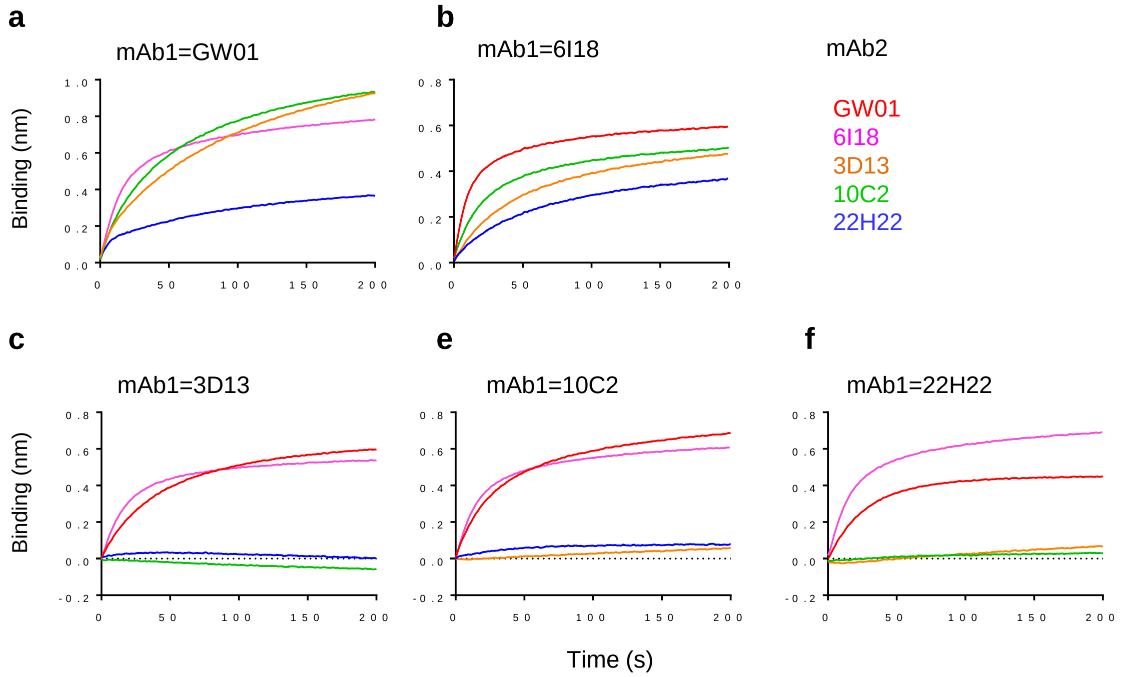

Fig. S3. Identification of binding epitope of mAbs as measured by biolayer interferometry competition assay.

Binding of (**a**) GW01, (**b**) 6I18, (**c**) 3D13, (**d**) 10C2, and (**e**) 22H22 to SARS-CoV-2 RBD in competition with other mAbs. mAb1 was immobilized on an anti-human IgG (AHC) biosensor and then incubated with SARS-CoV-2 RBD. The graph shows the shift in nanometres after the binding of mAb2 to mAb1-RBD complexes.

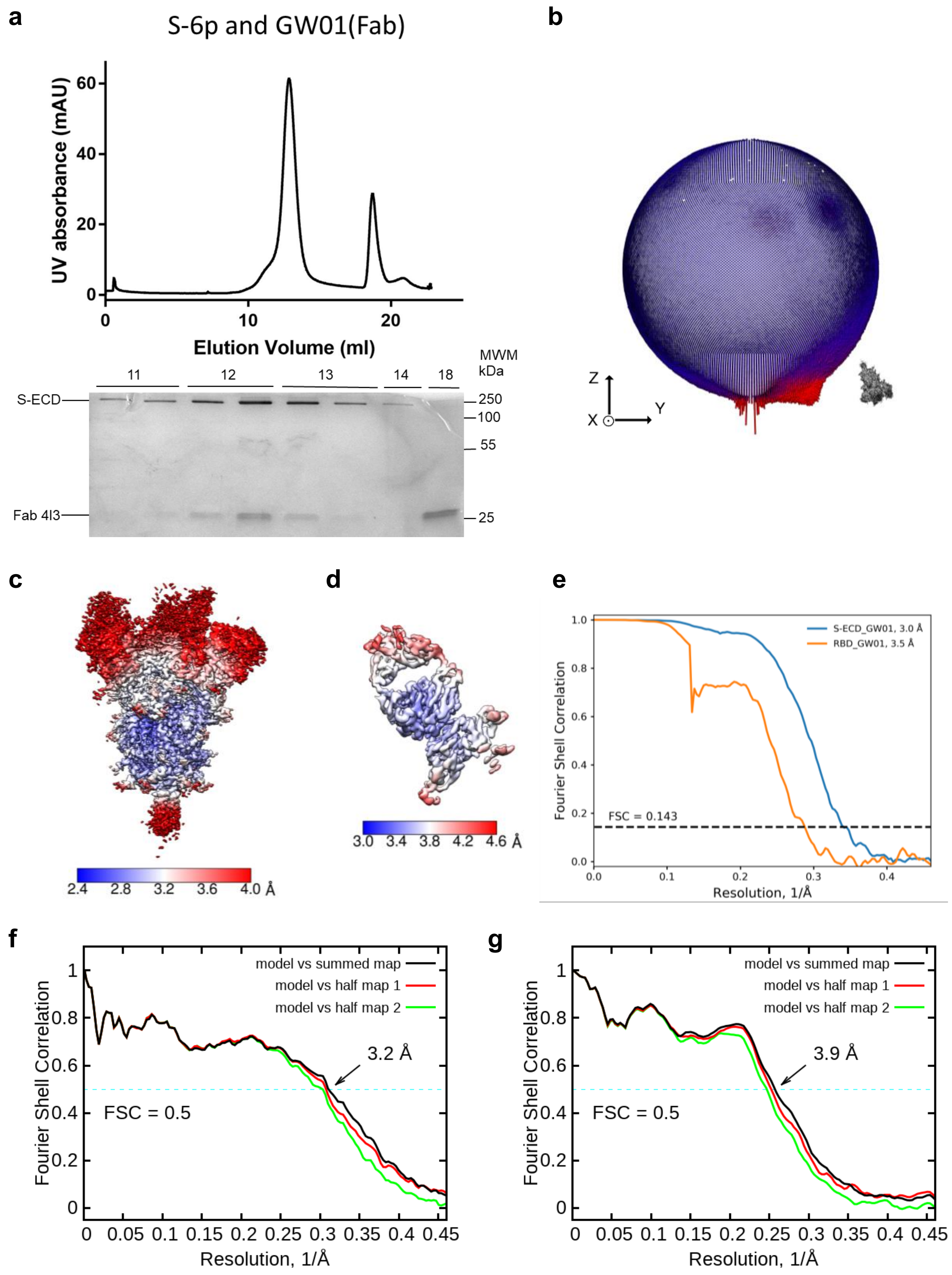

Fig. S4. Cryo-EM analysis of S-ECD in complex with GW01.

(a) Representative SEC purification profile of S-ECD in complex with GW01.

(**b**) Euler angle distribution in the final 3D reconstruction of S-ECD bound with

GW01. (**c**)-(**d**) Local resolution map for the 3D reconstruction of the overall structure and RBD-GW01 subcomplex, respectively. (**e**) FSC curve of the overall structure (blue) and RBD-GW01 subcomplex (orange). (**f**) FSC curve of the refined model of S-ECD bound with GW01 versus the overall structure against which it was refined (black); of the model refined against the first half map versus the same map (red); and of the model refined against the first half map versus the second half map (green). The small difference between the red and green curves indicates that the refinement of the atomic coordinates did not suffer from overfitting. (**g**) FSC curve of the refined model of the RBD-GW01 subcomplex, which is the same as (**f**).

a

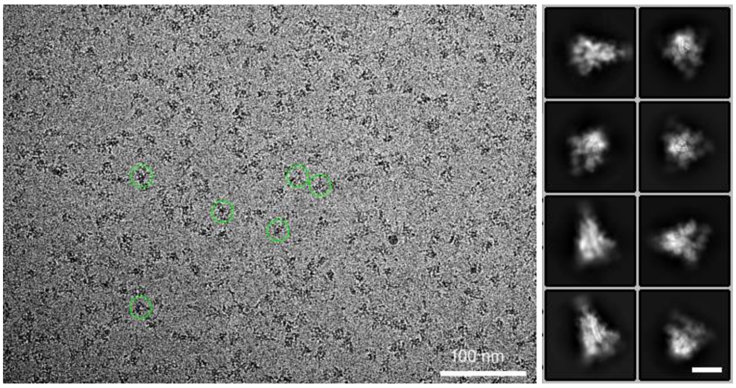
100 nm

b

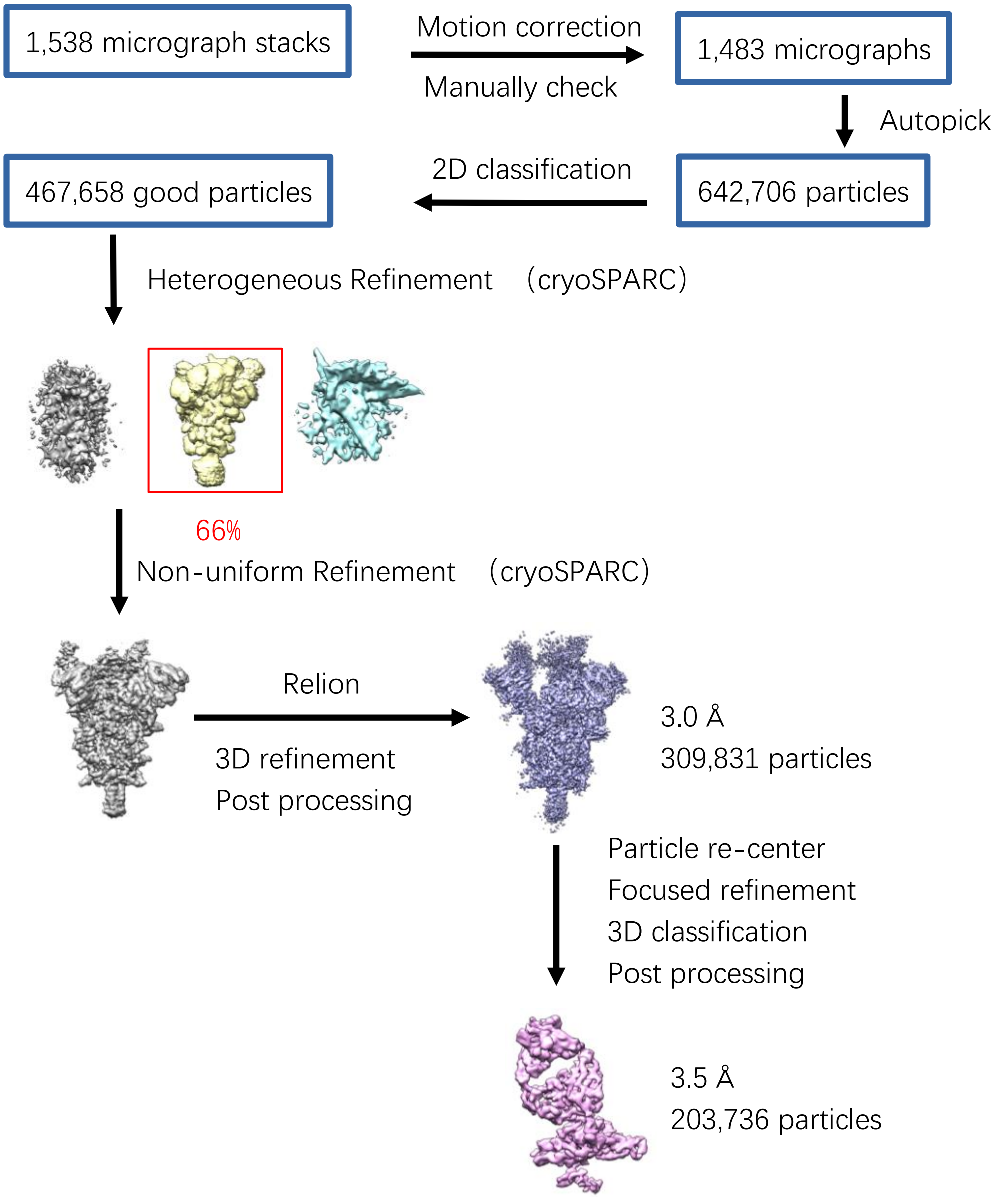
1,538 micrograph stacks
Motion correction
Manually check
1,483 micrographs
Autopick
642,706 particles
2D classification
467,658 good particles
Heterogeneous Refinement (cryoSPARC)
66%
Non-uniform Refinement (cryoSPARC)
Relion
3D refinement
Post processing
3.0 Å
309,831 particles
Particle re-center
Focused refinement
3D classification
Post processing
3.5 Å
203,736 particles

Fig. S5. Data processing of S-ECD in complex with GW01.

(**a**) Representative cryo-EM micrograph and 2D class averages of cryo-EM particle images of S-ECD bound with GW01. The scale bar in 2D class averages is 10 nm. (**b**) Flow chart of data processing. Please refer to the 'Data Processing' section in Methods for details.

a

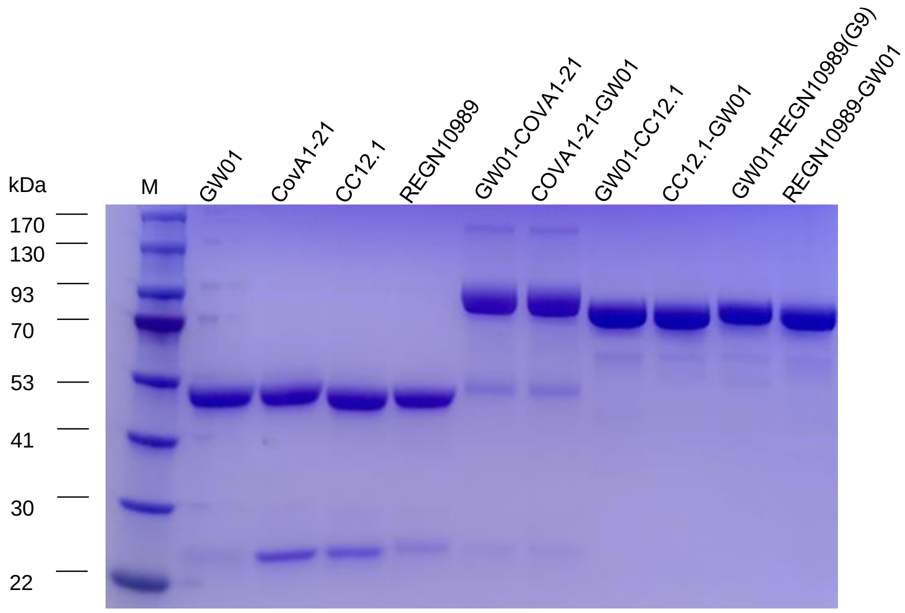

b

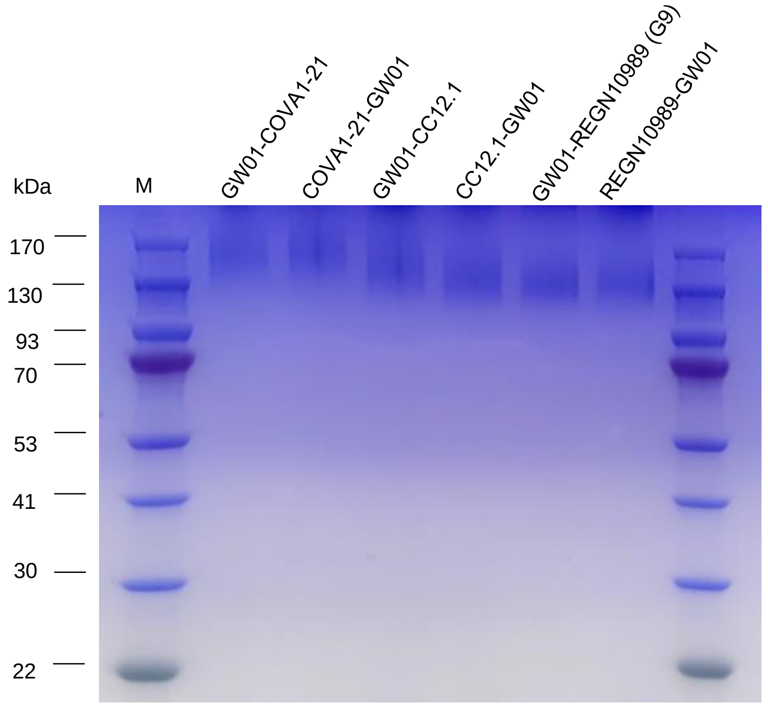

Fig. S6. Expression, purification and characterization of GW01-related bispecific antibodies.

(**a**) GW01-related bispecific antibodies were expressed by HEK293F cells and purified by protein G. Purity was detected by SDS–PAGE. GW01, COVA1-21 and CC12.1 were used as controls. (**b**) Crosslinking SDS–PAGE of GW01-related bispecific antibodies**.**

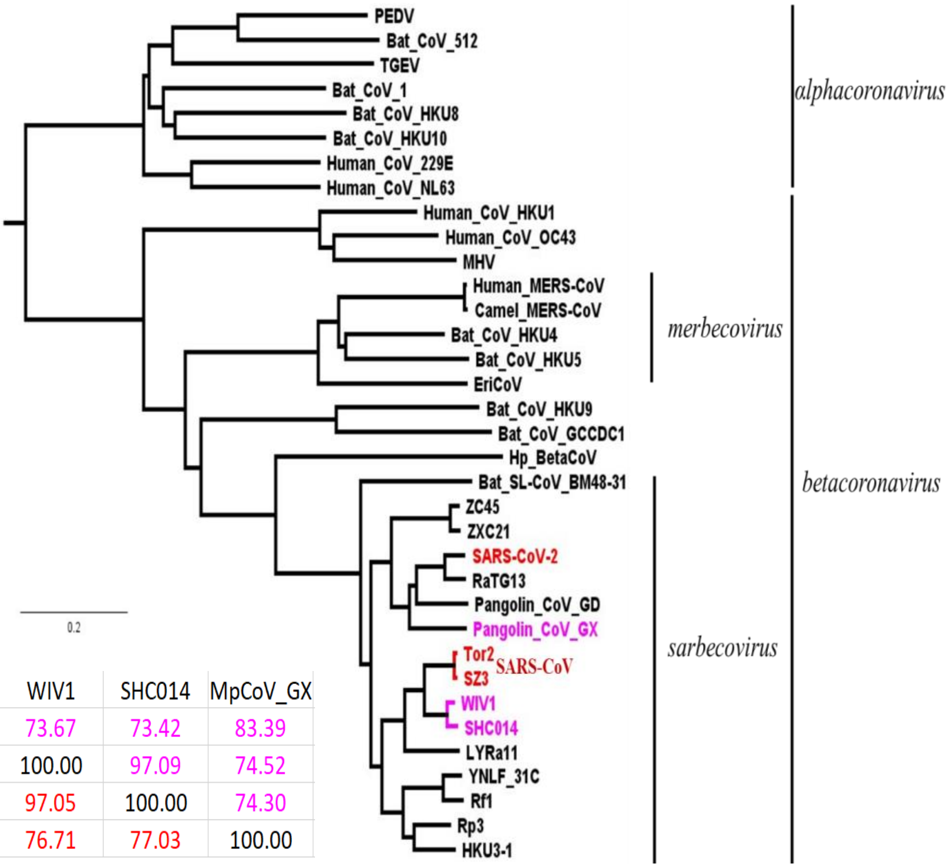

aa/nt	SARS-CoV-2	WIV1	SHC014	MpCoV_GX
SARS-CoV-2	100.00	73.67	73.42	83.39
WIV1	76.59	100.00	97.09	74.52
SHC014	76.74	97.05	100.00	74.30
MpCoV_GX	92.30	76.71	77.03	100.00

Fig. S7. Phylogenetic tree of sarbecoviruses.

SARS-CoV-2, BtSr-WIV1 and BtSr-SHC014, and pangolin-originated coronavirus-GX belong to the Sarbecovirus subgenus. The spike protein of pangolin CoV-GX is more closely related to the SARS-CoV-2 spike, with 83.39% nucleotide similarity and 92.30% amino acid similarity, while the two bat-origin viruses are more closely related to SARS-CoV, and their spike proteins share 76.59 and 76.74% amino acid identity with the SARS-CoV-2 spike, respectively.
